# Supplementary material for: Are transient protein-protein interactions more dispensable?
Source: PLoS Comput Biol. 2022 Apr 11;18(4):e1010013. doi: 10.1371/journal.pcbi.1010013 (PMC9000134; doi:10.1371/journal.pcbi.1010013)
Supplement: S1 Text — Fig A in S1 Text. Distribution of PPI transient classifications across multiple properties. Distribution of the number of times a PPI was classified as transient based on 8 different structural and biophysical measurements: PPI strength, transience in time (based on gene expression levels), transience in space (based on gene expression and promotor activity levels), stoichiometry in time (based on gene expression levels), stoichiometry in space (based on gene expression and promotor activity levels), and number of mutually exclusive PPIs. Fig B in S1 Text. Change in PPI binding free energy upon mutation. Change in PPI binding free energy (ΔΔG) distribution for all disease and common non-disease interfacial mutations in both structural interactomes Y2H-SI and Lit-SI. ΔΔG values were calculated using FoldX. Table A in S1 Text. Performance of predictions of temporally transient and permanent PPIs. Performance measures for predicting temporally transient PPIs and temporally permanent PPIs in human when benchmarked against experimental data combined from La et al. (2013) and Mintseris and Weng (2003). Table B in S1 Text. Dispensable content derived from different binding ΔΔG cut-offs. Dispensable content among weak and strong PPIs calculated using different binding ΔΔG cut-offs for predicting edgetic disruptions of PPIs in the two human structural interactomes Y2H-SI and Lit-SI. Table C in S1 Text. Edgetic mutation data derived from experiments. Number of common mutations and disease mutations in the experimental data of Sahni et al. 2015 that edgetically disrupt transient and permanent PPIs as defined by different biophysical and spatiotemporal properties. PPI disruptions by mutations were obtained from experiments of Sahni et al. 2015. PPI classifications were determined computationally. Table D in S1 Text. Dispensable content among transient and permanent PPIs in experiments. Edgotype probabilities for neutral and mildly deleterious mutations in the experimental data [file pcbi.1010013.s011.pdf]

Are transient protein-protein interactions more dispensable?

Supporting Information

Ghadie and Xia

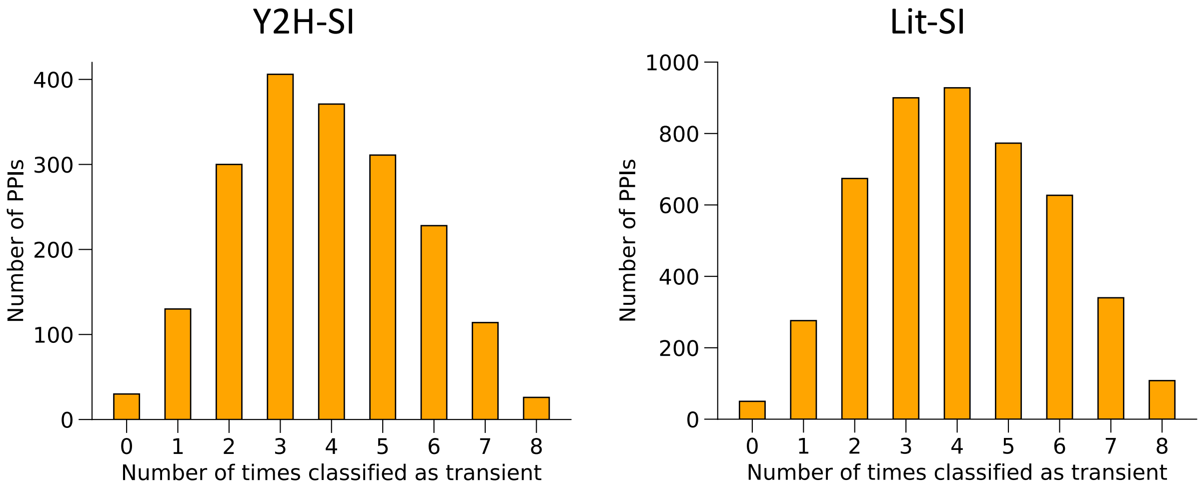

Fig A. Distribution of PPI transient classifications across multiple properties
Distribution of the number of times a PPI was classified as transient based on 8 different structural and biophysical measurements: PPI strength, transience in time (based on gene expression levels), transience in space (based on gene expression and promotor activity levels), stoichiometry in time (based on gene expression levels), stoichiometry in space (based on gene expression and promotor activity levels), and number of mutually exclusive PPIs.

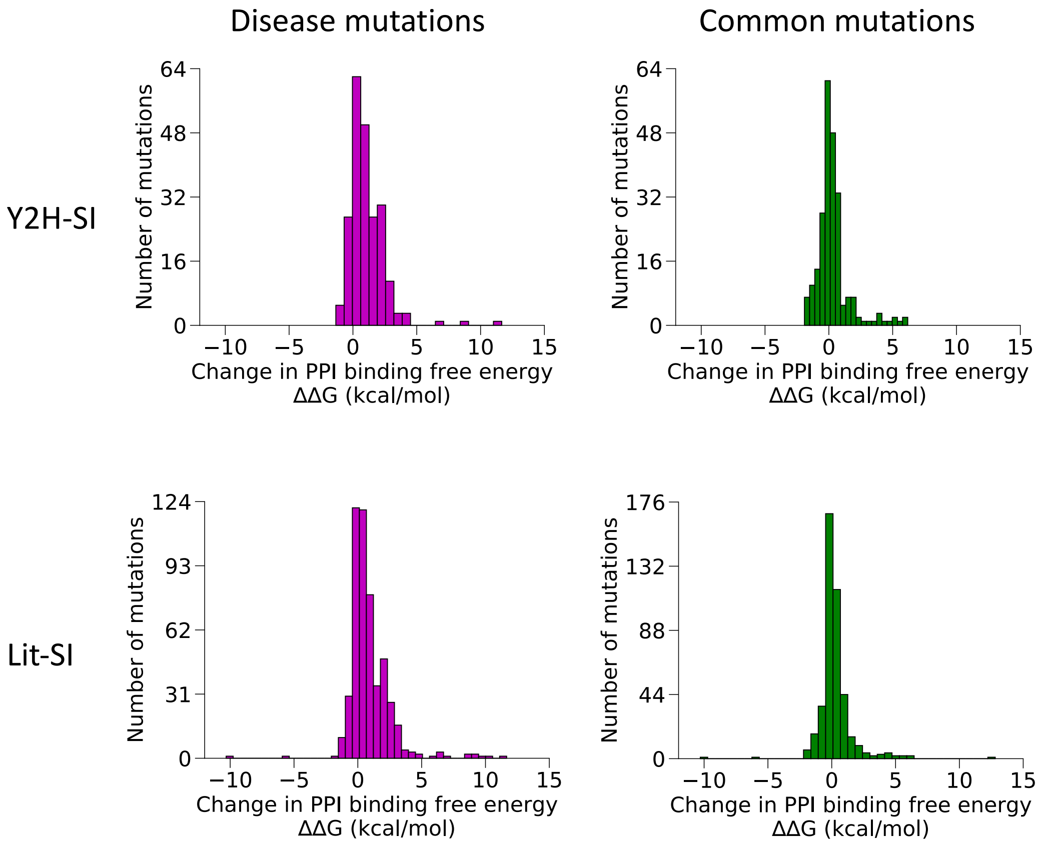

Fig B. Change in PPI binding free energy upon mutation

Change in PPI binding free energy (ΔΔG) distribution for all disease and common non-disease interfacial mutations in both structural interactomes Y2H-SI and Lit-SI. ΔΔG values were calculated using FoldX.

Table A. Performance of predictions of temporally transient and permanent PPIs
Performance measures for predicting temporally transient PPIs and temporally permanent PPIs in human when benchmarked against experimental data combined from La et al. (2013) and Mintseris and Weng (2003).

PPI properties		Prediction performance					
Transient PPIs	Permanent PPIs	Accuracy (balanced)	Precision	TPR	FPR	TPR / FPR	P-value
Transient in time	Permanent in time	70%	99%	0.51	0.1	5.1	0.019
Unbalanced over time	Balanced over time	56%	97%	0.41	0.3	1.4	0.74
Mutually exclusive	Simultaneously possible	78%	98%	0.95	0.4	2.4	1.1×10^{-5}

Table B. Dispensable content derived from different binding ΔΔG cut-offs
Dispensable content among weak and strong PPIs calculated using different binding ΔΔG cut-offs for predicting edgetic disruptions of PPIs in the two human structural interactomes Y2H-SI and Lit-SI.

	Interactome	Y2H-SI			Lit-SI		
	ΔΔG cut-off (kcal/mol)	1	2	3	1	2	3
Dispensable content (%)	Weak PPIs	2.7	5.2	0	7	3.7	7.4
	Strong PPIs	3.4	3.4	2.7	7.7	7.4	4.5
Confidence interval (95%)	Weak PPIs	0.3 – 18.5	0.6 – 34.4	Na	3.6 – 13.1	1.1 – 11.3	1.6 – 27.7
	Strong PPIs	1.4 – 7.8	1 – 10.8	0.3 – 18.5	4.5 – 12.9	3.8 – 13.8	1.4 – 13.6

Table C. Edgetic mutation data derived from experiments

Number of common mutations and disease mutations in the experimental data of Sahni et al. 2015 that edgetically disrupt transient and permanent PPIs as defined by different biophysical and spatiotemporal properties. PPI disruptions by mutations were obtained from experiments of Sahni et al. 2015. PPI classifications were determined computationally.

PPI properties			Common mutations			Disease mutations		
Transient PPIs	Permanent PPIs	Expression data	Total	Disrupt transient PPIs	Disrupt permanent PPIs	Total	Disrupt transient PPIs	Disrupt permanent PPIs
Transient in time	Permanent in time	Time-course (GEO)	47	0	2	190	18	37
Transient in space	Permanent in space	Tissue (Illumina)	46	1	0	183	22	26
		Tissue (Fantom5)	47	0	2	195	32	28
Unbalanced over time	Balanced over time	Time-course (GEO)	47	0	2	189	14	40
Unbalanced over space	Balanced over space	Tissue (Illumina)	47	0	2	186	21	30
		Tissue (Fantom5)	47	0	2	195	30	30

Table D. Dispensable content among transient and permanent PPIs in experiments
Edgotype probabilities for neutral and mildly deleterious mutations in the experimental data of Sahni et al. 2015 calculated among transient and permanent PPIs directly from Table C, assuming that common mutations are effectively neutral (N) and that disease mutations are mildly deleterious (M) on average. The resulting dispensable contents P(N|T) and P(N|P) among both transient (T) and permanent (P) PPIs were calculated using Bayes' theorem. Columns represent the following, P(T|N): probability (%) for neutral mutations (N) to edgetically disrupt transient PPIs (T), P(T|M): probability (%) for mildly deleterious mutations (M) to edgetically disrupt transient PPIs (T), P(N|T): dispensable content among transient PPIs defined as the probability (%) for transient PPIs to be effectively neutral upon disruption, P(P|N): probability (%) for neutral mutations (N) to edgetically disrupt permanent PPIs (P), P(P|M): probability (%) for mildly deleterious mutations (M) to edgetically disrupt permanent PPIs (P), P(N|P): dispensable content among permanent PPIs defined as the probability (%) for permanent PPIs to be effectively neutral upon disruption. CI: 95% confidence interval (%) for dispensable contents P(N|T) and P(N|P). PPI disruptions by mutations were obtained from experiments of Sahni et al. 2015. PPI classifications were determined computationally.

PPI properties			Transient PPIs					Permanent PPIs				
Transient PPIs	Permanent PPIs	Expression data	P(T|N)	P(T|M)	$\frac{P(T\|N)}{P(T\|M)}$	P(N|T)	CI	P(P|N)	P(P|M)	$\frac{P(P\|N)}{P(P\|M)}$	P(N|P)	CI
Transient in time	Permanent in time	Time-course (GEO)	0	9.5	0	0	Na	4.3	19.5	0.22	10	2.7 – 30.8
Transient in space	Permanent in space	Tissue (Illumina)	2.2	12	0.18	8.4	1.3 – 40	0	14.2	0	0	Na
		Tissue (Fantom5)	0	16.4	0	0	Na	4.3	14.4	0.30	13.1	3.6 – 37.9
Unbalanced over time	Balanced over time	Time-course (GEO)	0	7.4	0	0	Na	4.3	21.2	0.20	9.3	2.5 – 29.0
Unbalanced over space	Balanced over space	Tissue (Illumina)	0	11.3	0	0	Na	4.3	16.1	0.27	11.9	3.2 – 35.2
		Tissue (Fantom5)	0	15.4	0	0	Na	4.3	15.4	0.28	12.4	3.4 – 36.3

Table E. Mono-edgetic mutation data obtained from predictions

Number of common mutations and disease mutations that edgetically disrupt a single transient PPI or permanent PPI defined by different structural, biophysical and spatiotemporal properties in the two human structural interactomes Y2H-SI and Lit-SI.

PPI properties				Common mutations			Disease mutations		
Transient PPIs	Permanent PPIs	Expression data	SI	Total	Disrupt a transient PPI	Disrupt a permanent PPI	Total	Disrupt a transient PPI	Disrupt a permanent PPI
Weak	Strong		Y2H	1,080	2	6	348	4	21
			Lit	2,867	24	22	1,572	39	39
Transient in time	Permanent in time	Time-course (GEO)	Y2H	1,080	5	3	347	17	7
			Lit	2,867	15	31	1,571	51	26
Transient in space	Permanent in space	Tissue (Illumina)	Y2H	1,079	3	4	341	12	6
			Lit	2,863	19	23	1,569	52	23
		Tissue (Fantom5)	Y2H	1,080	2	6	346	17	6
			Lit	2,862	18	23	1,572	42	36
Unbalanced over time	Balanced over time	Time-course (GEO)	Y2H	1,080	1	7	347	8	16
			Lit	2,867	11	35	1,571	30	47
Unbalanced over space	Balanced over space	Tissue (Illumina)	Y2H	1,080	6	2	347	17	7
			Lit	2,866	27	18	1,571	40	37
		Tissue (Fantom5)	Y2H	1,080	5	3	346	15	8
			Lit	2,862	20	21	1,572	32	46
1-4 mutually exclusives	No mutually exclusives		Y2H	1,080	3	2	348	17	8
			Lit	2,867	28	10	1,572	31	34
≥5 mutually exclusives			Y2H	1,080	3		348	0	
			Lit	2,867	8		1,572	13	

Table F. Dispensable content among transient and permanent PPIs based on mono-edgetic mutations

Edgotype probabilities for neutral and mildly deleterious mutations calculated directly from edgotype numbers in Table E, assuming that common mutations are effectively neutral (N) and that disease mutations are mildly deleterious (M) on average. The resulting dispensable contents P(N|T) and P(N|P) among both transient (T) and permanent (P) PPIs were calculated using Bayes' theorem. Columns represent the following, SI: structural interactome, P(T|N): probability (%) for neutral mutations (N) to edgetically disrupt a single transient PPI (T), P(T|M): probability (%) for mildly deleterious mutations (M) to edgetically disrupt a single transient PPI (T), P(N|T): dispensable content among transient PPIs defined as the probability (%) for transient PPIs to be effectively neutral upon disruption, P(P|N): probability (%) for neutral mutations (N) to edgetically disrupt a single permanent PPI (P), P(P|M): probability (%) for mildly deleterious mutations (M) to edgetically disrupt a single permanent PPI (P), P(N|P): dispensable content among permanent PPIs defined as the probability (%) for permanent PPIs to be effectively neutral upon disruption.

PPI properties				Transient PPIs				Permanent PPIs			
Transient PPIs	Permanent PPIs	Expression data	SI	P(T|N)	P(T|M)	$\frac{P(T\|N)}{P(T\|M)}$	P(N|T)	P(P|N)	P(P|M)	$\frac{P(P\|N)}{P(P\|M)}$	P(N|P)
Weak	Strong		Y2H	0.2	1.1	0.18	7.6	0.6	6.0	0.10	4.5
			Lit	0.8	2.5	0.32	14.7	0.8	2.5	0.32	13.6
Transient in time	Permanent in time	Time-course (GEO)	Y2H	0.5	4.9	0.10	4.6	0.3	2.0	0.15	6.6
			Lit	0.5	3.2	0.16	7.6	1.1	1.7	0.65	25
Transient in space	Permanent in space	Tissue (Illumina)	Y2H	0.3	3.5	0.09	3.9	0.4	1.8	0.22	9.7
			Lit	0.7	3.3	0.21	9.3	0.8	1.5	0.53	21.8
		Tissue (Fantom5)	Y2H	0.2	4.9	0.04	1.9	0.6	1.7	0.35	14
			Lit	0.6	2.7	0.22	10.7	0.8	2.3	0.35	15.2
Unbalanced over time	Balanced over time	Time-course (GEO)	Y2H	0.1	2.3	0.04	2	0.6	4.6	0.13	6.7
			Lit	0.4	1.9	0.21	9.3	1.2	3.0	0.40	17.2
Unbalanced over space	Balanced over space	Tissue (Illumina)	Y2H	0.6	4.9	0.12	5.5	0.2	2.0	0.10	4.5
			Lit	0.9	2.5	0.36	15.9	0.6	2.4	0.25	12
		Tissue (Fantom5)	Y2H	0.5	4.3	0.12	5.2	0.3	2.3	0.13	5.8
			Lit	0.7	2.0	0.35	14.9	0.7	2.9	0.24	11.3
1-4 mutually exclusives	No mutually exclusives		Y2H	0.3	4.9	0.06	2.8	0.2	2.3	0.09	3.9
			Lit	1.0	2.0	0.50	20.2	0.3	2.2	0.14	7.6
≥5 mutually exclusives			Y2H	0.3	0	Na	Na				
			Lit	0.3	0.8	0.37	14.7				

Table G. Confidence intervals for dispensable content based on mono-edgetic mutations
95% confidence intervals for estimates of dispensable content P(N|T) and P(N|P) in Table F based on mono-edgetic mutations.

PPI properties				95% confidence interval	
Transient PPIs	Permanent PPIs	Expression data	SI	P(N|T)	P(N|P)
Weak	Strong		Y2H	1.5 – 30.9	1.9 – 10.3
			Lit	9.4 – 22.2	8.6 – 20.9
Transient in time	Permanent in time	Time-course (GEO)	Y2H	1.8 – 11.5	1.8 – 21.2
			Lit	4.4 – 12.7	16.6 – 35.8
Transient in space	Permanent in space	Tissue (Illumina)	Y2H	1.1 – 12.4	3 – 27.4
			Lit	5.7 – 14.7	13.6 – 33.2
		Tissue (Fantom5)	Y2H	0.4 – 7.6	5 – 33.5
			Lit	6.5 – 17.2	9.6 – 23.1
Unbalanced over time	Balanced over time	Time-course (GEO)	Y2H	0.3 – 14	2.9 – 14.7
			Lit	4.9 – 16.9	11.9 – 24.3
Unbalanced over space	Balanced over space	Tissue (Illumina)	Y2H	2.2 – 12.7	1 – 18.3
			Lit	10.4 – 23.4	7.2 – 19.2
		Tissue (Fantom5)	Y2H	2 – 12.9	1.6 – 18.7
			Lit	9.1 – 23.4	7.1 – 17.6
1-4 mutually exclusives	No mutually exclusives		Y2H	0.8 – 8.9	0.9 – 16.1
			Lit	13.2 – 29.5	3.9 – 14.2
≥5 mutually exclusives			Y2H	Na	
			Lit	6.7 – 29.3	
