# Supplementary material for: Are transient protein-protein interactions more dispensable?
Source: PLoS Comput Biol. 2022 Apr 11;18(4):e1010013. doi: 10.1371/journal.pcbi.1010013 (PMC9000134; doi:10.1371/journal.pcbi.1010013)
Supplement: Attachment — Submitted filename: PLOSCOMPUBIOLREVIEW.pdf [file pcbi.1010013.s012.pdf]

Journal: PLOS Computational Biology
Manuscript ID : PCOMPBIOL-D-21-01091
Title : Are transient protein-protein interactions more dispensable?
Author(s): Mohamed Ghadie and Yu Xia

The goal of the manuscript is to determine if transient PPI are as important as permanent PPI for the cell function and the cell evolution (first two sentences of abstract). To address the question, the authors assess the dispensable content of transient and permanent PPI, dispensable being defined as neutral upon mutation. They use two human interactomes and build structural interactomes equivalent using PDB and structural homology modeling to assess the residue composition of every PPI interface of the interactomes. Then they use a set of mutations known to be common or disease –related and their fold X in silico generation to determine the PPI interfaces containing mutations in their interactomes. They use Fold X to compute free energy changes upon mutation and combining energy and interface mutation features they determine which mutation is edgetic, defined in the manuscript as having a mutation in a PPI interface and a free energy change >0,5 kcalmol-1.
Finally they use different methods to sort the interactomes into transient and permanent PPI classes and apply the Bayes theorem to calculate the dispensable content in each case. The comparison between permanent and transient PPI reveals similar dispensable contents for both PPI regardless the mean used to class the interactomes into transient and permanent PPIs.
The authors conclude that transient PPI are as important as permanent PPI for cell function and cell evolution (lines 460 to 466).

There are several issues with the manuscript, which needs to be solved before re-submission/publication. The manuscript is heavy and difficult to follow, it would profit from simplification and the details about all the calculation need to be provided. A lot of the work is in ref 16 so the authors should focused on what is new and make clear what was done before to avoid thinking everything has been done in this work. For example the structural interactomes were done in ref 16 and that is not clear when reading. Nevertheless, in the present manuscript there are more PPI for each interactome than in ref 16, so that could be commented. Fold X was not used in ref 16, so that could also be highlighted as new and adding a new constraint to identify edgetic mutations with a backup of free energy calculations. But I could not find the data on the ΔΔG calculated from mutants generated in silico with Fold X I suppose. It will be interesting to compare precisely the experimental results (ref4) with the present study as Fold X does not reproduce the impact of real mutations so its limit can be discussed and revealed different type of mutation impact (identify with Fold X or not identify with Fold x).

Please below see some points to be accounted for by the authors before re-submission.

Comments

Conceptual issues/definition:

The 'long standing debate' on the importance of transient versus permanent PPI for cell function and cell evolution needs to be backed up with references. Many transient PPIs are extremely fundamental for cells, p53 for example to say the least, so this issue needs to be supported.

It is not clear why being neutral upon mutations means dispensable, does that mean that PPI robust to mutation do not contribute to the interactomes and can be disposed of? Why can't they be on the contrary the only PPI remaining functionally resistant when the interactomes undergo mutation perturbations?

There are some confusion on the definition of neutral, is neutral defining functionally neutral? or no impact on the edges of the interactomes (edgotype)?

line 188 : common mutations are assumed neutral and disease-related mutation are assumed mildly deleterious : so neutral is here defined in terms of functional impact of the mutation and not in terms of impacting the interactomes PPI, right?
On figure 2: N (=neutral) are mutations that do not disturb any PPI so that is among all common and disease-related mutations those who do not modify the PPI (ie the interface amino acid type, ie the edgicity) ? So this neutral does not refer to the common mutation because according to suptable 2 most mutations do not impact any PPI (ie edgicity) regardless common or disease-related (in Y2H-SI 4% (47/1081) and 39% (135/348) of common and disease-related mutations are impacting PPI).
On figure 1: in the formula the N stands for Neutral is that in terms of edgicity ?
On lines 163 to 171 : edgicity is computed for common and disease related mutations and the authors conclude on neutral upon mutation so neutral in terms of edgicity.

The authors assess dispensable as being edgetic but neutral functionally (if I have understood properly from the calculation, e.g. line 203 : 7%) so neutral needs to be defined precisely to avoid the confusion along the text. Moreover, a change of amino acid type in an interface does not imply loss of the interface/interaction as introduced in ref 33 for edgetic, and the choice of $\Delta\Delta G$>0,5 kcal.mol-1 for loosing the interface needs to be justified and backed up with references. **Where are the $\Delta\Delta$Gs?**
So the definition of neutral should be clarified as well as the definition of dispensable :
In text line 41 or line 60, the definition of dispensable is 'dispensable, i.e., effectively neutral upon disruption'.
In line 60 for the permanent PPI we find :'While it is widely assumed that permanent PPIs carry out important cellular functions and are mostly indispensable'. Do we conclude that indispensable means functionally robust to mutation?

Calculations:

The authors have to provide the details of the calculation: how did they get 0.3%, 1.2%, 10.3%, etc. The easiest would be to have a table for each method of transient/permanent classification that summarizes all the data use for the calculations.

Line 167 : relative frequency : is the ratio Tc/Td (see table 1) a relative frequency? It is the ratio of two percentages so it has no unit, it is a factor, a propensity. For example, D_T=Td/Tc = 6.6 (Table 1, Y2H weak/strong set) means the disease-related mutations have a propensity to be edgetic around 7-times more than common mutations.

Role of the different Transient/Permanent classification

The argument and input of using several methods to classify Transient and Permanent PPIs is unclear. Moreover, the classification changes but not the mutations or the method to calculate the dispensable content, hence once the authors find that both transient and permanent exhibit similar dispensable content, re-shuffling the classification is not going to change much, is it? In particular since the number of PPI concerned by mutation edgetic impact is very small (e.g. 47 in Y2H-SI common and 135 in Y2H disease-related frm SupTable 2) so maybe there are little differences in the final data used to calculate. That will be seen if the authors provide a table with the mutation data use to calculate the dispensable content for each Transient/Permanent classification.

Miscellaneous comments

The authors should be careful with the vocabulary : they use non synonymous but new missense

mutations, they use strong and weak and transient and permanent. The work needs to be clear and accessible to all readers, not just to experts who are versed with using different words for the same notion.

I am not sure when I read legend of figure 2 what the authors are referring to for the 'disrupt any PPI in the human interactome as determined by experiments? There are no experiments in the manuscript and the authors refer to another paper (ref 4) and we understand that line 222. The data of the ref 4 is added to figure 2 but there is no explanation how they calculated them or whether the calculations where performed in ref 4. Ref 4 should be indicated on the legend of figure 2. The result of ref 4 and how it compares to the results of the authors should be discussed not just mentioned as being consistent. This comparison is already discussed in ref 16, but here they use FoldX and classification of transient and permanent not found in ref 16 so it will be interesting to discuss again.

The formula should not be integrated in the text but should appear on a separate line and given a number.

line 199 : P(EW|S) is assumed to be approximately 0.
=> this seems to be by definition rather than an approximation. The authors assume strongly detrimental mutations (S) to be quasi null which is by definition mutations disturbing all PPI due to an overall protein stabillity issue (see lines145 and ref 4) and therefore not edgetic. Nevertheless line 157 in suptable 2 in Y2H disease related mutations is an example of a mutation that destabilizes all PPI (only 1 in column perturbations) so is that edgetic or quasi null ?

Supmat 2 does not provide information on the free energy changes but on the mutations (common and disease-related) that modify one residue type on a PPI.
Supdata3 provides the WT ΔG but I do not find the ΔΔG for mutations, data needed to select edgetic mutations and compute the dispensable content. The readers should not need to extract the information from supmaterial to compute the dispendable content. In fact the figures are over processed data and the supmat contained all rough data, a compromise between the two needs to be shown as figures in the manuscript.

The authors talk about edgotype 'predictions' (lines 146 to 150) but isn't it more like a definition? First, there are no experimental evidences that the mutations impact the PPI (or that is discuss in ref 4 and therefore the authors should mention it). In ref 33, edgetic is defined as loss of interactions, but in the table the mutation has modified the type of residue at an interfacial position and affected the ΔΔG by 0,5 kcalmol-1 (data not found) : is the fold X 0,5 kcalmol-1 enough to claim a loss of binding/interactions ?

Widely used method appears ten times in the manuscript sometimes more than once in the same sentence. A method widely used does not guarantee its accuracy and does not change its limits. Fold X is used but has limitations compared to real experiments so only some impact can be 'assumed' using FoldX. The authors may want to backup the reliability of the free energy calculation using Fold X with references comparing experimental and fold X results. The authors can also explain that given the data, Fold X is a good approximation, but acknowledge that when experiments are performed things might be different.

In discussion

The authors discuss the high quality of their approach by referring to their own work and at the same time mentioning widely used methods with no references, so maybe it will re-enforce their results if they refer to colleagues using for example Fold X and validating it the free energy calculation compared to experiments.

lines: Finally, our study reveals the power of structural and systems biology computations in elucidating the phenotypic effects of interactome network perturbations by genetic mutations.
=> This link between genotype and phenotype relies on Fold X in silico mutations and free energy calculations, PDB structural modeling, change in interfaces with no experimental validations, and many assumptions (interface mutation and free energy changes for edgetic assessment, neutral are common, mildly deleterious are disease-related). The authors could discuss the limit of Fold X and discuss ref 4 results to show to what extent a 'computational approach' is performing well compared to experiment. Are there experimental mutations in PPI of the two interactomes that do not show edgetic output but were predicted to do so by Fold X (or just by assuming a mutation at interface modify the binding)? Are there experimental mutations in PPI of the two interactomes that are edgetic as predicted by Fold-X? As Fold X does not reproduce an experimental mutation, it is expected to find discrepancy between experiments and computational assessment of the dispensable PPI.
