# Supplementary material for: Are transient protein-protein interactions more dispensable?
Source: PLoS Comput Biol. 2022 Apr 11;18(4):e1010013. doi: 10.1371/journal.pcbi.1010013 (PMC9000134; doi:10.1371/journal.pcbi.1010013)
Supplement: Attachment — Submitted filename: Response_to_reviewers.pdf [file pcbi.1010013.s013.pdf]

RESPONSE TO COMMENTS FROM REVIEWER #1

<u>Reviewer's Comments:</u> (1) The goal of the manuscript is to determine if transient PPIs are as important as permanent PPIs for the cell function and the cell evolution (first two sentences of abstract). To address the question, the authors assess the dispensable content of transient and permanent PPIs, dispensable being defined as neutral upon mutation. They use two human interactomes and build structural interactomes equivalent using PDB and structural homology modeling to assess the residue composition of every PPI interface of the interactomes. Then they use a set of mutations known to be common or disease–related and their FoldX in silico generation to determine the PPI interfaces containing mutations in their interactomes. They use FoldX to compute free energy changes upon mutation and combining energy and interface mutation features they determine which mutation is edgetic, defined in the manuscript as having a mutation in a PPI interface and a free energy change >0.5 kcal.mol^{-1}. Finally they use different methods to sort the interactomes into transient and permanent PPI classes and apply the Bayes' theorem to calculate the dispensable content in each case. The comparison between permanent and transient PPIs reveals similar dispensable contents for both PPIs regardless of the mean used to classify the interactomes into transient and permanent PPIs. The authors conclude that transient PPIs are as important as permanent PPIs for cell function and cell evolution (lines 460 to 466).

There are several issues with the manuscript, which needs to be solved before re-submission/publication. The manuscript is heavy and difficult to follow, it would profit from simplification and the details about all the calculation need to be provided. A lot of the work is in ref 16 so the authors should focused on what is new and make clear what was done before to avoid thinking everything has been done in this work. For example the structural interactomes were done in ref 16 and that is not clear when reading. Nevertheless, in the present manuscript there are more PPIs for each interactome than in ref 16, so that could be commented. FoldX was not used in ref 16, so that could also be highlighted as new and adding a new constraint to identify edgetic mutations with a backup of free energy calculations. But I could not find the data on the ΔΔG calculated from mutants generated in silico with FoldX I suppose. It will be interesting to compare precisely the experimental results (ref 4) with the present study as FoldX does not reproduce the impact of real mutations so its limit can be discussed and revealed different type of mutation impact (identified with FoldX or not identified with FoldX). Please below see some points to be accounted for by the authors before re-submission.

<u>Author's Response:</u> We thank the reviewer for the positive evaluation of our work and for the very constructive comments which helped us revise and significantly improve our manuscript. We have addressed all reviewer comments in detail as described below in our responses to other comments. In particular, we provided more details about our calculations in the Results section, and highlighted what is new in our work compared to what has been done in our previous work as well as other work in the literature. We also provided our mutation ΔΔG results predicted by FoldX in the supplementary material, and validated our FoldX-based predictions against experimental data. In addition, we included a thorough and detailed discussion of the strengths and limitations of our computational approach and validated it against multiple experimental and computational datasets. More details are provided in our responses to other reviewer comments below.

<u>Reviewer's Comments:</u> (2) Comments - Conceptual issues/definition: The 'long standing debate' on the importance of transient versus permanent PPIs for cell function and cell evolution needs to be backed up with references. Many transient PPIs are extremely fundamental for cells, p53 for example to say the least, so this issue needs to be supported.

<u>Author's Response:</u> We thank the reviewer for the insightful and important comment which helped us improve our manuscript. Indeed, as the reviewer mentioned, many transient protein-protein

interactions (PPIs) are fundamental to the cell. For example, transient PPIs have been found to play important roles in defining the structure of interactome networks, such as regulating interactome modularity (Han *et al.* 2004 *Nature* 430:88-93; Perkins *et al.* 2010 *Structure* 18:1233-43) and guiding the formation of obligate protein complexes (Blundell *et al.* 2006 *Nature* 444:279-80; Tang *et al.* 2006 *Nature* 444:383-6). Other transient PPIs are known to participate in multiple cellular pathways and biochemical processes, including secretory pathways (Nyfeler *et al.* 2005 *PNAS* 102:6350), signal transduction (Sungkaworn *et al.* 2017 *Nature* 550:543-7; Thomas *et al.* 2004 *Nature Rev. Neurosci.* 5:173-83; Kholodenko 2006 *Nature Rev. Mol. Cell Biol.* 7:165-76), immune response (Liu *et al.* 2007 *Nature Rev. Immunol.* 7:202-12), chaperone-guided protein folding (Wu *et al.* 2019 *Nature Commun.* 10:4833; Rosenzweig *et al.* 2019 *Nature Rev. Mol. Cell Biol.* 20:665-80), apoptosis (Moll *et al.* 2005 *Curr. Opin. Cell Biol.* 17:631-6), and tumor suppression (Bieging *et al.* 2014 *Nature Rev. Cancer* 14:359-70). While these studies show that many transient PPIs play important roles in cellular function and human disease, it remains unclear whether this observed functional significance generalizes to most transient PPIs.

On the other hand, transient PPIs differ from permanent PPIs in their structural composition and binding dynamics (Acuner Ozbabacan *et al.* 2011 *Prot. Engin. Design Selec.* 24:635-48; Perkins *et al.* 2010 *Structure* 18:1233-43; Vishwanath *et al.* 2017 *Curr. Opin. Struc. Biol.* 44:77-86). They tend to occur among certain protein hubs, known as “date hubs”, which interact with multiple partners in a mutually exclusive manner using the same binding interface (Han *et al.* 2004 *Nature* 430:88-93; Kim *et al.* 2006 *Science* 314:1938). This behaviour is contrary to that of permanent PPIs which tend to occur among a different type of protein hubs, known as “party hubs”, which interact with multiple partners simultaneously using multiple binding interfaces (Han *et al.* 2004 *Nature* 430:88-93; Kim *et al.* 2006 *Science* 314:1938). Mutually exclusive transient PPIs are often mediated through short linear motifs that typically occur in intrinsically disordered regions (Perkins *et al.* 2010 *Structure* 18:1233-43; Cumberworth *et al.* 2013 *Biochem. J.* 454:361-9; Tompa *et al.* 2014 *Mol. Cell* 55:161-9). These binding motifs tend to be smaller in surface area and contain less hydrophobic residues than interfaces of permanent PPIs, thus they bind with weaker affinities (Perkins *et al.* 2010 *Structure* 18:1233-43). Linear motifs also evolve very rapidly (Tompa *et al.* 2014 *Mol. Cell* 55:161-9; Perkins *et al.* 2010 *Structure* 18:1233-43), contributing in part to the higher rate of rewiring among transient PPIs compared to permanent PPIs (Mintseris *et al.* 2005 *Proteins: Struc. Func. Bioinf.* 53:629-39; Beltrao *et al.* 2007 *PLOS Comput. Biol.* 3:e25; Sun *et al.* 2012 *PLOS Comput. Biol.* 8:e1002411; Studer *et al.* 2016 *Science* 354:229). Indeed, empirical studies in phospho-proteomics and molecular evolution estimate that as much as ~65% of transient phosphorylation sites in yeast are unconstrained under evolution and may have no important function (Landry *et al.* 2009 *Trends Genet.* 25:193-7; Levy *et al.* 2012 *Phil. Trans. Roy. Soc. B Biol. Sci.* 367:2594-606).

Therefore, taking these observations all together, we chose to replace our previous statement “it remains widely debated whether transient PPIs are equally important” with the following statement “it remains an open question whether transient PPIs have more dispensable content than other permanent PPIs (Landry *et al.* 2009 *Trends Genet.* 25:193-7; Levy *et al.* 2012 *Phil. Trans. Roy. Soc. B Biol. Sci.* 367:2594-606; Studer *et al.* 2016 *Science* 354:229)”. We believe that this new statement better reflects the current state of the literature.

We have included this discussion of current literature on transient PPIs in the Introduction section (Pages 5-6 Lines 68-94).

<u>Reviewer's Comments:</u> (3) It is not clear why being neutral upon mutations means dispensable, does that mean that PPI robust to mutation do not contribute to the interactomes and can be disposed of? Why can't they be on the contrary the only PPI remaining functionally resistant when the interactomes undergo mutation perturbations?

<u>Author's Response:</u> We thank the reviewer for the very insightful and important comment and for giving us the opportunity to further clarify our work. We wish to clarify that our definition of dispensable PPI is not "neutral upon mutation". Rather, our definition of dispensable PPI is "neutral upon disruption of PPI by mutation". We agree with the reviewer that the phrase "neutral upon mutation" is ambiguous with two completely different interpretations: (i) "neutral upon disruption of PPI by mutation", which is the subject of our current study and our definition of dispensable PPI; or (ii) "neutral due to robustness of PPI against mutation", which is a completely different phenomenon and beyond the scope of our current study. In our study, we make a clear distinction between PPIs that are effectively neutral upon disruption by mutations on the one hand, and PPIs that are robust to the presence of mutations at the binding interface hence are not disrupted by the mutation on the other hand. As the reviewer mentioned, many PPIs may be robust to the presence of mutations at the binding interface in the sense that those PPIs are not disrupted by the mutation, i.e., they are not eliminated from the interactome as a result of mutation (Vo *et al.*, *Cell* 164:310-323, 2016; Leducq *et al.*, *PLoS Genet.* 8:e1003161, 2012; Ghadie *et al.*, *Curr. Opin. Struc. Biol.* 50:42-48, 2018). However, these robust PPIs are beyond the scope of our current study. On the other hand, our definition of dispensable PPIs refers to a completely different type of phenomenon. Dispensable PPIs are those PPIs that are disrupted in the presence of a mutation at the interface, i.e., they are completely eliminated from the interactome as a result of mutation, however their elimination from the interactome has no measurable deleterious impact on organismal fitness (Ghadie and Xia 2019 *Nature Commun.* 10:3205). We have revised our manuscript accordingly to include this important clarification in the Introduction section (Pages 4-5 Lines 61-67).

<u>Reviewer's Comments:</u> (4) There are some confusion on the definition of neutral, is neutral defining functionally neutral? or no impact on the edges of the interactomes (edgotype)? line 188 : common mutations are assumed neutral and disease-related mutations are assumed mildly deleterious : so neutral is here defined in terms of functional impact of the mutation and not in terms of impacting the interactomes PPI, right? On figure 2: N (=neutral) are mutations that do not disturb any PPI so that is among all common and disease-related mutations those who do not modify the PPI (i.e. the interface amino acid type, i.e. the edgicity)? So this neutral does not refer to the common mutation because according to supplementary table 2 most mutations do not impact any PPI (i.e. edgicity) regardless common or disease-related (in Y2H-SI 4% (47/1081) and 39% (135/348) of common and disease-related mutations are impacting PPI). On figure 1: in the formula the N stands for Neutral is that in terms of edgicity? On lines 163 to 171: edgicity is computed for common and disease related mutations and the authors conclude on neutral upon mutation so neutral in terms of edgicity. The authors assess dispensable as being edgetic but neutral functionally (if I have understood properly from the calculation, e.g. line 203 : 7%) so neutral needs to be defined precisely to avoid the confusion along the text.

<u>Author's Response:</u> We thank the reviewer for pointing out this source of confusion and for giving us the opportunity to better clarify our work. As the reviewer explained, we have mistakenly used the label 'N' in our study to refer to two different effects that a mutation may have. In terms of fitness effect, a mutation can be either neutral, mildly deleterious or strongly detrimental. In terms of impact on PPIs, a mutation can be either edgetic if it disrupts a PPI by disrupting the binding interface or non-edgetic if it does not disrupt PPI interface, regardless of whether it is effectively

neutral or deleterious to fitness. Both effectively neutral mutations and deleterious mutations may or may not disrupt PPIs, thus they both may or may not be edgetic.

In the manuscript text, and in Figure 1, we used the term neutral (abbreviated as N) to refer to mutations that have a neutral (non-deleterious) effect on fitness, i.e., functionally neutral as the reviewer chose to describe them, regardless of how they impact PPIs. This definition of neutral (N), i.e., neutral in fitness effect (or functionally neutral), is what we refer to in our assumption "that common mutations from healthy individuals are effectively neutral and that disease mutations are mildly deleterious on average" (Page 11 Lines 194-196). This definition of neutral (neutral in fitness effect) is also what the label 'N' refers to in Figure 1 and in all the manuscript text. However, we mistakenly used the same label 'N' in Figures 2-5 to also refer to mutations that do not disrupt any PPI, regardless of the mutation's fitness effect or associated phenotype (i.e. common or disease-causing). To remove this source of confusion in the revised manuscript, we have replaced the label 'N' for mutations that do not disrupt any PPI in Figures 2-5 by the label 'D'.

Therefore, in the revised version of the manuscript, the term "neutral" and its abbreviation 'N' always refer to neutral in terms of fitness effect (or functionally neutral as described by the reviewer) regardless of how the mutation impacts the interactome (edgetic or not edgetic), whereas the label 'D' in Figures 2-5 refers to mutations that do not disrupt any PPI in the human interactome regardless of the phenotype associated with the mutation (common or disease-causing). Consequently, PPIs that are effectively neutral upon disruption are those whose edgetic disruption by mutation has no deleterious effect on organismal fitness, i.e. they are functionally neutral upon disruption. We have revised our manuscript accordingly to include the correct labels for mutations that do not disrupt PPIs in Figures 2-5, and also include this clarification on the meanings of the terms "neutral" and "completely dispensable PPIs" in the manuscript text (Pages 4-5 Lines 61-67; Page 11 Lines 197-200; Figures 2-5).

Reviewer's Comments: (5) Moreover, a change of amino acid type in an interface does not imply loss of the interface/interaction as introduced in ref 33 for edgetic, and the choice of ΔΔG > 0.5 kcal.mol^{-1} for losing the interface needs to be justified and backed up with references.

Author's Response: We thank the reviewer for the very important and constructive comment which helped us significantly improve our manuscript. As the reviewer mentioned, not every mutation at the binding interface results in PPI disruption. Our structure-based edgotype prediction approach predicts that a mutation at the binding interface edgetically disrupts the PPI if and only if it causes a change in binding free energy ΔΔG > 0.5 kcal/mol. This ΔΔG cut-off has been used and proven to provide high-quality predictions of PPI disruption in previous structural biology studies (Zhao *et al.* 2014 *PLOS Comput. Biol.* 10:e1003592; Cui *et al.* 2019 *Genes* 10:933; Ghadie and Xia 2019 *Nature Commun.* 10:3205; Ghadie and Xia 2021 *Front. Bioinf.* 1:690769).

In our recent study in particular (Ghadie and Xia 2021 *Front. Bioinf.* 1:690769), we have validated our FoldX-based edgotype prediction method on the experimental data of Sahni *et al.* 2015 (*Cell* 161:647-60) using a ΔΔG cut-off of 0.5 kcal/mol for predicting PPI disruption. Out of 23 mutations that were found to be edgetic in experiments, 7 were correctly predicted by our method to be edgetic, giving a true positive rate (TPR) of 0.3. On the other hand, out of 57 mutations that were found to be non-edgetic by experiments, only 2 were incorrectly predicted by our method to be edgetic, giving a false positive rate (FPR) of 0.04. A TPR that is equal to FPR indicates that predictions are no better than random expectations. Our TPR is 7.5 times larger than our FPR ($p = 0.002$, two-sided Fisher's exact test), indicating that our FoldX-based edgotype

prediction method and choice of ΔΔG cut-off for edgetic mutations is very high quality. In addition, our Bayesian framework for estimating dispensable content has also been shown to be robust to different choices of ΔΔG cut-off close to 0.5 kcal/mol, particularly 0.3 and 0.7 kcal/mol (Ghadie and Xia 2021 *Front. Bioinf.* 1:690769), further proving that a ΔΔG cut-off in the neighborhood of 0.5 kcal/mol is highly reliable.

We have revised our manuscript accordingly to include this justification of ΔΔG cut-off in the Discussion section (Page 34 Lines 636-641; Pages 35-36 Lines 672-681).

<u>Reviewer's Comments:</u> (6) Where are the ΔΔGs?

<u>Author's Response:</u> We thank the reviewer for pointing this out. Indeed, we forgot to include mutation ΔΔG values in our first submission. Thus, in our second submission we have included a list of all mutation ΔΔG values that were used in our study as Supplementary Dataset 3. Table legends can be found on Page 57 of the revised manuscript.

<u>Reviewer's Comments:</u> (7) So the definition of neutral should be clarified as well as the definition of dispensable: In text line 41 or line 60, the definition of dispensable is 'dispensable, i.e., effectively neutral upon disruption'. In line 60 for the permanent PPI we find: 'While it is widely assumed that permanent PPIs carry out important cellular functions and are mostly indispensable'. Do we conclude that indispensable means functionally robust to mutation?

<u>Author's Response:</u> We thank the reviewer for the very important and constructive comment. As the reviewer mentioned and as explained in our previous responses to reviewer comments, a mutation can be either neutral, mildly deleterious or strongly detrimental in terms of fitness effect. In terms of impact on PPIs, a mutation can be either edgetic if it disrupts a PPI by disrupting the binding interface or non-edgetic if it does not disrupt PPI interface, regardless of whether it is effectively neutral or deleterious to fitness. Both effectively neutral mutations and deleterious mutations may or may not disrupt PPIs, thus they both may or may not be edgetic.

Furthermore, while many PPIs may be robust to the presence of mutations at the binding interface in the sense that they are not disrupted by the mutation (i.e., they are not eliminated from the interactome in the presence of mutation), our definition of completely dispensable PPIs refers to a different type of phenomenon. Completely dispensable PPIs are those that are disrupted in the presence of a mutation at the interface (i.e., they are completely eliminated from the interactome in the presence of mutation), however their elimination from the interactome has no measurable deleterious impact on organismal fitness in terms of function and phenotype. Accordingly, indispensable PPIs are those whose complete disruption (i.e., elimination from the interactome as a result of mutation) is deleterious to cellular and organismal fitness in terms of function and phenotype.

We have revised our manuscript accordingly to include this clarification on the meanings of the terms "neutral" and "completely dispensable PPIs" in the manuscript text (Pages 4-5 Lines 61-67; Page 11 Lines 197-200). In addition, to remove any source of confusion on the definition of dispensability in the revised version of the manuscript, we have replaced our previous statement "While it is widely assumed that permanent PPIs carry out important cellular functions and are mostly indispensable, it remains widely debated whether transient PPIs are equally important" with the following statement "It remains an open question whether transient PPIs have more dispensable content than other permanent PPIs" (Page 5 Lines 68-69).

Reviewer's Comments: **(8) Calculations: The authors have to provide the details of the calculation: how did they get 0.3%, 1.2%, 10.3%, etc. The easiest would be to have a table for each method of transient/permanent classification that summarizes all the data used for the calculations.**

Author's Response: We thank the reviewer for the very important comment which helped us revise and improve our manuscript. The probabilities for effectively neutral mutations (N) to edgetically disrupt weak transient PPIs (T): P(T|N) = 3/1080 = 0.3% and to edgetically disrupt strong permanent PPIs (P): P(P|N) = 13/1080 = 1.2%, as well as the probabilities for mildly deleterious mutations (M) to edgetically disrupt weak transient PPIs (T): P(T|M) = 7/348 = 2% and to edgetically disrupt strong permanent PPIs (P): P(P|M) = 36/348 = 10.3% were all calculated directly from our edgotype predictions for common mutations and disease-causing mutations, assuming that common mutations are effectively neutral (N) and that disease-causing mutations are mildly deleterious (M) on average.

We agree with the reviewer that it is important to provide the detailed calculation for these probabilities. As suggested by the reviewer, in the revised manuscript, we have included the detailed calculation of dispensable content among weak transient PPIs and among strong permanent PPIs in the yeast two-hybrid structural interactome (Y2H-SI) in the Results section as an example calculation (Pages 11-14 Lines 201-254). All other calculations of dispensable content throughout the manuscript follow the exact same calculation provided in the Results section for weak and strong PPIs. In addition, we have also included a detailed description of our Bayesian framework for calculating dispensable content in the Methods section (Pages 46-47 Lines 902-923).

Furthermore, as suggested by the reviewer, we have provided a table (Table 1) that summarizes all the mutation edgotype data that was used to calculate the dispensable content among each type of PPIs in the two structural interactomes Y2H-SI and Lit-SI (Page 26). We have also provided a second table (Table 2) that summarizes all the probabilities for effectively neutral mutations and mildly deleterious mutations to disrupt transient PPIs and permanent PPIs, for each type of PPI classification in Y2H-SI and Lit-SI (Page 27). These probabilities in Table 2 were calculated directly from Table 1 by dividing the number of mutations that disrupt a certain type of PPI by the total number of mutations as listed in Table 1. This calculation is also shown in our example calculation in the Results section (Pages 11-14 Lines 201-254). Table 2 also includes the dispensable content calculated for transient PPIs and permanent PPIs based on each type of PPI classification described in the text.

Reviewer's Comments: **(9) Line 167: relative frequency: is the ratio Tc/Td (see table 1) a relative frequency? It is the ratio of two percentages so it has no unit, it is a factor, a propensity. For example, DT = Td/Tc = 6.6 (Table 1, Y2H weak/strong set) means the disease-related mutations have a propensity to be edgetic around 7-times more than common mutations.**

Author's Response: We thank the reviewer for the insightful comment. To improve clarity, we have replaced the notations T_c and T_d in Table 2 (Page 27) (previously Table 1 in our first submission) with the notations P(T|N) and P(T|M), respectively, since the values of T_c and T_d represent the probabilities for common mutations (assumed to be neutral (N)) and disease-causing mutations (assumed to be mildly deleterious (M)) to disrupt transient PPIs (T), respectively. Similarly, we have replaced the notations P_c and P_d in Table 2 (Page 27) with the notations P(P|N) and P(P|M), respectively, since the values of P_c and P_d represent the probabilities for common mutations (assumed to be neutral (N)) and disease-causing mutations (assumed to be mildly deleterious (M)) to disrupt permanent PPIs (P), respectively. These new notations in Table 2 are

now consistent with the notations used in our example calculation of dispensable content described in the Results section (Pages 11-14 Lines 201-254), and also consistent with the notations used in our framework for dispensable content calculation described in the Methods section (Pages 46-47 Lines 902-923). In addition, as suggested by the reviewer, we have replaced the term "relative frequency" in the manuscript text with the term "propensity" as reference to the ratio T_c/T_d, which is now P(T|N)/P(T|M) in Table 2 of the revised manuscript (Page 11 Lines 206-209).

Reviewer's Comments: (10) Role of the different Transient/Permanent classifications: The argument and input of using several methods to classify Transient and Permanent PPIs is unclear. Moreover, the classification changes but not the mutations or the method to calculate the dispensable content, hence once the authors find that both transient and permanent exhibit similar dispensable content, re-shuffling the classification is not going to change much, is it? In particular since the number of PPI concerned by mutation edgetic impact is very small (e.g. 47 in Y2H-SI common and 135 in Y2H disease-related from Supplementary Table 2) so maybe there are little differences in the final data used to calculate. That will be seen if the authors provide a table with the mutation data used to calculate the dispensable content for each Transient/Permanent classification.

Author's Response: We thank the reviewer for the very important and constructive comment which helped us significantly improve our manuscript. Detecting transient PPIs in experiments and through computational predictions is very challenging (Acuner Ozbabacan *et al.* 2011 *Prot. Engin. Design Selec.* 24:635-48; Schilder *et al.* 2013 *Curr. Opin. Struct. Biol.* 23:911-8; Vishwanath *et al.* 2017 *Curr. Opin. Struct. Biol.* 44:77-86). With different limitations and biases associated with experimental techniques for detecting PPIs (Cafarelli *et al.* 2017 *Curr. Opin. Struct. Biol.* 44:201-10; Wodak *et al.* 2013 *Curr. Opin. Struct. Biol.* 23:941-53), large-scale datasets of transient PPIs are not available. However, databases that curate PPI sequence and structural information such as linear motifs (Kumar *et al.* 2020 *Nucleic Acids Res.* 48:D296-306; Grant *et al.* 2011 *Bioinf.* 27:1017-8), domain-motif interactions (Obenauer *et al.* 2003 *Nucleic Acids Res.* 31:3635-41), domain-domain interactions (Mosca *et al.* 2014 *Nucleic Acids Res.* 42:D374-9), three-dimensional protein structures (Berman *et al.* 2003 *Nature Struct. Mol. Biol.* 10:980), and binding affinities (Vreven *et al.* 2015 *J. Mol. Biol.* 427:3031-41) from both experimental and computational studies can be used to predict transient and permanent PPIs. Some studies have used PPI binding affinity measurements from experiments to identify transient and permanent interactions (Vreven *et al.* 2015 *J. Mol. Biol.* 427:3031-41; La *et al.* 2013 *Proteins: Struct. Func. Bioinf.* 81:805-18). The scope of these studies is limited by the small number of PPIs with affinity data available from experiments. Computational studies have used interface structural information (Mintseris *et al.* 2003 *Proteins: Struct. Func. Bioinf.* 53:629-39; Kim *et al.* 2006 *Science* 314:1938), protein sequence information (Ofran *et al.* 2003 *J. Mol. Biol.* 325:377-87) and machine learning models (Block *et al.* 2006 *Proteins: Struct. Func. Bioinf.* 65:607-22; Park *et al.* 2009 *BMC Bioinf.* 10:36) to predict transient and permanent interactions. Other studies made use of gene expression data which does not rely on experimentally-solved protein structures (Jansen *et al.* 2002 *Genome Res.* 12:37-46; Bossi *et al.* 2009 *Mol. Syst. Biol.* 5:260). Since the criteria and information that were used to detect transient interactions vary among these studies, each study has its own biases and sources of error. Moreover, with the large increase in PPI datasets recently mapped by experiments in human (Luck *et al.* 2017 *Trends Biochem. Sci.* 42:342-54; Luck *et al.* 2020 *Nature* 580:402-8), and given the difficulty in identifying transient PPIs in experiments, there is a great need for new computational efforts to accurately classify transient and permanent interactions in these new datasets, taking into account different structural and biophysical properties that distinguish transient PPIs from permanent PPIs.

Our study provides a comprehensive list of transient PPIs and permanent PPIs in human, characterized using diverse structural and biophysical properties. These properties include, among others, the temporal and spatial co-expression of interaction partners, a property that has not been explored enough in recent literature especially on large-scale PPI datasets such as the HuRI dataset. While discrete properties such as the number of mutually exclusive PPIs sharing the same binding interface can be strong indicators of the transient or permanent nature of interaction, other properties such as binding affinity and the spatial and temporal co-expression of interaction partners may vary for some PPIs over a continuum of values overlapping between transient PPIs and permanent PPIs. For example, while transient PPIs tend to have weak binding affinities, it may be that some of them have stronger binding affinities closer to those of permanent PPIs. At the same time, while transient PPIs tend to have low co-expression levels and unbalanced stoichiometries between interaction partners, some of them may have higher co-expression levels and more balanced stoichiometries closer to those of permanent PPIs. Hence, there is no perfect borderline that separates transient PPIs from permanent PPIs based on such properties. Our study addresses this limitation by considering different structural, biophysical and spatiotemporal properties for classifying transient and permanent PPIs. These properties produce different sets of PPI classifications that are diverse enough to capture the aforementioned variabilities in PPI properties, but also with significant overlap (Supplementary Dataset 5). On average, a PPI is classified as transient by ~4 out of 8 properties in both Y2H-SI and Lit-SI, with 36% of PPIs in Y2H-SI and 40% of PPIs in Lit-SI classified as transient by 5 or more properties (Figure S1 in Supplementary Information). This diversity in PPI classifications results in a noticeable change across different PPI properties in the proportions P(T|N), P(T|M), P(P|N) and P(P|M) of neutral (N) and mildly deleterious (M) mutations that edgetically disrupt transient (T) and permanent (P) PPIs (Tables 1 and 2, Pages 26-27). Nonetheless, the two ratios of proportions P(T|N)/P(T|M) and P(P|N)/P(P|M) are less affected by such changes (Table 2, Page 27), resulting in estimates of dispensable PPI content that are broadly consistent across all PPI properties (<~20%) (Table 2). These results highlight the importance of considering different biophysical measurements in our study for classifying transient PPIs and permanent PPIs.

We have revised our manuscript accordingly to include this very important justification and discussion for using different properties to classify transient PPIs and permanent PPIs in the Introduction section (Pages 6-7 Lines 95-113) and Discussion section (Pages 29-30 Lines 520-546).

<u>Reviewer's Comments:</u> (11) Miscellaneous comments: The authors should be careful with the vocabulary: they use non synonymous but new missense mutations, they use strong and weak and transient and permanent. The work needs to be clear and accessible to all readers, not just to experts who are versed with using different words for the same notion.

<u>Author's Response:</u> We thank the reviewer for the important suggestion. As suggested by the reviewer, we have replaced the term "non-synonymous mutations" in the revised version of the manuscript with the term "missense mutations". In addition, we agree with the reviewer that using the terms "weak PPIs" and "strong PPIs" to describe transient PPIs and permanent PPIs may be confusing for the reader. Therefore, we have replaced the terms "weak PPIs" and "strong PPIs" in the revised version of the manuscript with the terms "weak transient PPIs" and "strong permanent PPIs", respectively (Pages 10-14 Lines 180-265).

Reviewer's Comments: (12) I am not sure when I read the legend of figure 2 what the authors are referring to for the 'disrupt any PPI in the human interactome as determined by experiments? There are no experiments in the manuscript and the authors refer to another paper (ref 4) and we understand that line 222. The data of the ref 4 is added to figure 2 but there is no explanation how they calculated them or whether the calculations where performed in ref 4. Ref 4 should be indicated on the legend of figure 2.

Author's Response: We thank the reviewer for the very important and constructive comment. As the reviewer pointed out, the fractions of common mutations and disease mutations that edgetically disrupt any PPI in the human interactome as determined by experiments (shown in the pie charts in panel b of Figure 2) were obtained from the experimental study of Sahni *et al.* (*Cell* 161:647-60) and were not calculated in our study. These fractions were then used to calculate the fraction of dispensable PPIs for experiments shown in panel c of Figure 2, using our Bayesian framework described in the Methods section. Therefore, to avoid any confusion we have added a reference to the experimental study of Sahni *et al.* in the legend of Figure 2 as suggested by the reviewer, and also added a reference beside the pie charts for experimental edgotypes in panel b (Page 12 Lines 216-223; Figure 2).

Reviewer's Comments: (13) The result of ref 4 and how it compares to the results of the authors should be discussed not just mentioned as being consistent. This comparison is already discussed in ref 16, but here they use FoldX and classification of transient and permanent is not found in ref 16 so it will be interesting to discuss again.

Author's Response: We thank the reviewer for the very important and constructive comment which helped us revise and improve our manuscript. First, we would like to clarify that in our previous study (Ghadie and Xia 2019 *Nature Commun.* 10:3205), two different prediction methods were used to predict edgetic and non-edgetic mutations. The first method was the gemoetry-based method, which does not use FoldX calculations of change in binding free energy (ΔΔG) upon mutation to predict PPI disruption by mutation. This geometry-based approach is described in the Results section of that study under the heading "Geometry-based prediction of mutation edgotypes". The second prediction method used in that study is the physics-based method, which predicts PPI disruption by mutation based on FoldX calculations of mutation ΔΔG. This physics-based approach is described in the Results section of that study under the heading "Physics-based calculation of dispensable PPI content". The ΔΔG cut-off that was used in the physics-based approach of that study for predicting PPI disruption by mutation is the same cut-off used in our current study, 0.5 kcal/mol. It is worth noting here that in our current study we also estimated dispensable content among all PPIs in the human interactome using two PPI datasets, the HuRI dataset mapped by systematic yeast two-hybrid (Y2H) screens (Luck *et al.* 2020 *Nature* 580:402-8) and the literature-curated dataset from the IntAct database. Our estimates in the current study are consistent with our estimates from our previous study (Ghadie and Xia 2019 *Nature Commun.* 10:3205) which were derived from much smaller datasets, the HI-II-14 dataset also mapped by systematic Y2H screens (Rolland *et al.* 2014 *Cell* 159:1212-26) and an earlier version of literature-curated PPIs from the IntAct database. Furthermore, these estimates are also consistent with our estimates of dispensable content in the overall human interactome derived from mutation edgotypes obtained from experiments of Sahni *et al.* (*Cell* 161:647-60).

Nevertheless, as the reviewer mentioned, in our current study we also estimated dispensable content among transient PPIs and permanent PPIs, which was not done in our previous study of Ghadie and Xia 2019 nor in the experiments of Sahni *et al.* 2015. Therefore, to compare

our computational results with experiments, we estimated dispensable content for both transient PPIs and permanent PPIs, this time using mutation edgotypes obtained from experiments of Sahni *et al.* (*Cell* 161:647-60). We first classified PPIs in the experimental dataset as either transient or permanent, in both time and space, as well as balanced or unbalanced in stoichiometry over both time and space, following the same procedures we used to classify PPIs in our two structural interactomes Y2H-SI and Lit-SI. It is worth noting here too the very small size of the experimental dataset which consists of only 47 common mutations and 197 disease mutations, with only 2 common mutations and 62 disease mutations that are edgetic. Next, we calculated the fractions of mutations that are edgetic among transient PPIs (transient in time, transient in space, and unbalanced in stoichiometry) and the fraction of mutations that are edgetic among permanent PPIs (permanent in time, permanent in space, and balanced in stoichiometry), using edgotype data from experiments. Similar to our calculations in Y2H-SI and Lit-SI, we found that transient and permanent PPIs are both less likely to be disrupted by neutral mutations than by deleterious mutations (Tables S3 and S4 in Supplementary Information). We then estimated dispensable content among both transient PPIs and permanent PPIs using these edgotype results derived from experiments. Overall, our estimates are all below ~20%, consistent with our estimates obtained from predictions in Y2H-SI and Lit-SI, albeit with larger confidence intervals due to small sample size. These results derived from experimental mutation edgotypes further prove the high quality of our predicted mutation edgotypes and dispensable content estimates obtained from these predictions. A similar calculation of dispensable content among weak PPIs and strong PPIs and among mutually exclusive PPIs and simultaneously possible PPIs in experiments of Sahni *et al.* was not possible due to lack of structural data for the vast majority of PPIs that are disrupted by mutations in these experiments.

We have revised our manuscript accordingly to include this very important comparison between computational results and experiments (Pages 28-29 Lines 506-519; Pages 36-37 Lines 682-703).

Reviewer's Comments: (14) The formula should not be integrated in the text but should appear on a separate line and given a number.

Author's Response: We thank the reviewer for the constructive comment which helped us improve our manuscript. As suggested by the reviewer, we have moved all equations in the Results section and the Methods section to separate lines. We also gave each equation in the Methods section a number, which was also used in the Results section as a reference to the corresponding equation (Pages 13-14 Lines 226-254; Pages 46-47 Lines 902-923).

Reviewer's Comments: (15) Line 199: P(EW|S) is assumed to be approximately 0. => this seems to be by definition rather than an approximation. The authors assume strongly detrimental mutations (S) to be quasi null which is by definition mutations disturbing all PPI due to an overall protein stability issue (see lines 145 and ref 4) and therefore not edgetic. Nevertheless line 157 in supplementary table 2 in Y2H disease related mutations is an example of a mutation that destabilizes all PPIs (only 1 in column perturbations) so is that edgetic or quasi null?

Author's Response: We thank the reviewer for the insightful comment which helped us revise and improve our manuscript. As the reviewer mentioned, in our calculations we assume that strongly detrimental mutations (S) are predominantly quasi-null rather than edgetic. Hence, the probability for strongly detrimental mutations to be edgetic among both transient PPIs and permanent PPIs is

assumed to be approximately 0. The reviewer correctly points out that, by definition, quasi-null mutations are defined as mutations that disrupt the overall stability of the protein, leading to the loss of all PPIs associated with that protein. That being said, it remains an assumption (albeit a very reasonable one) that strongly detrimental mutations are predominantly quasi-null rather than edgetic. For example, while it is conceivable that some edgetic mutations are strongly detrimental (e.g., important edgetic mutations in essential proteins such as p53), it is reasonable to assume that most strongly detrimental mutations are quasi-null rather than edgetic, since it is much easier to create a strongly detrimental phenotype by disrupting the overall stability of the protein than by disrupting a specific binding interface.

In our first submission, we had used the notation E_W to refer to mutations that edgetically disrupt weak transient PPIs, and used the notation E_S to refer to mutations that edgetically disrupt strong permanent PPIs. However, for the sake of being consistent in our choice of notations throughout the manuscript and to avoid any confusion to the reader, in the revised version of the manuscript we have replaced the notation E_W with the notation T to refer to mutations that edgetically disrupt weak transient (T) PPIs. Similarly, we have replaced the notation E_S with the notation P to refer to mutations that edgetically disrupt strong permanent (P) PPIs (Pages 11-14 Lines 201-254). These two new notations for mutations that disrupt transient or permanent PPIs are now consistent throughout the manuscript text. Thus, in the revised version of the manuscript, we have replaced the statement "$P(E_W|S)$ is assumed to be approximately 0" with the new statement "$P(T|S)$ is assumed to be approximately 0" (Page 13 Line 232). Similarly, we have replaced the statement "$P(E_S|S)$ is assumed to be approximately 0" with the new statement "$P(P|S)$ is assumed to be approximately 0" (Page 14 Line 249).

Regarding mutations that disrupt more than one PPI, our structure-based prediction method makes a clear distinction between edgetic mutations and quasi-null mutations. Our method predicts a mutation to be edgetic if and only if it disrupts PPI by disrupting the binding interface. Quasi-null mutations on the other hand disrupt all PPIs by disrupting overall protein stability, thus creating other complex cellular and phenotypic changes that cannot be explained by simple PPI disruption. It is important to note here that each PPI disruption listed in Supplementary Dataset 2 is an edgetic disruption that was predicted by our method based on the change in binding free energy ($\Delta\Delta G$) created by the mutation at the binding interface. It is indeed possible for some edgetic mutations to disrupt more than one PPI (or all PPIs) by disrupting the binding interface if these disrupted PPIs share the same interface, and the mutation mentioned by the reviewer is an example of such mutations. In other words, even though this mutation disrupts all PPIs, it does so by locating on the surface of the protein and disrupting a specific binding interface mediating all PPIs, rather than by locating in the core of the protein and disrupting the overall stability of the protein. Hence, this mutation is still considered edgetic (rather than quasi-null).

Our calculations of dispensable PPI content assume that each edgetic mutation disrupts only one PPI, which is true for most edgetic mutations in Y2H-SI (50% of common mutations, and 58% of disease mutations) and in Lit-SI (82% of common mutations, and 66% of disease mutations). Nonetheless, to check whether our estimates of dispensable content are robust to the existence of edgetic mutations that disrupt more than one PPI, we repeated our calculations of dispensable content among both transient PPIs and permanent PPIs, this time replacing the fraction of mutations that are edgetic among common mutations and among disease mutations with the fraction of mutations that are mono-edgetic, i.e., those that disrupt only one PPI. Our results show that dispensable content among both transient PPIs and permanent PPIs remains below ~20% (Tables S5 and S6 in Supplementary Information), albeit with larger confidence intervals in some

cases due to smaller sample size for mono-edgetic mutations (Table S7 in Supplementary Information). These results prove that our estimates of dispensable content are robust to the existence of edgetic mutations that disrupt more than one PPI. We have revised our manuscript accordingly to include this important discussion in the Discussion section (Pages 37-38 Lines 704-726).

Reviewer's Comments: (16) Supplementary table 2 does not provide information on the free energy changes but on the mutations (common and disease-related) that modify one residue type on a PPI. Supplementary table 3 provides the WT ΔG but I do not find the ΔΔG for mutations, data needed to select edgetic mutations and compute the dispensable content. The readers should not need to extract the information from supplementary material to compute the dispensable content. In fact the figures are over processed data and the supplementary material contains all rough data, a compromise between the two needs to be shown as figures in the manuscript.

Author's Response: We thank the reviewer for the constructive comment which helped us revise and improve our manuscript. Indeed, we forgot to include mutation ΔΔG values in our first submission. Thus, in our revised submission, we have included a list of all mutation ΔΔG values that were used in our study as Supplementary Dataset 3. Table legends can be found on Page 57 of the revised manuscript. It should be noted here that the edgetic PPI disruptions and non-disruptions that were predicted for each mutation from the ΔΔG values provided in Supplementary Dataset 3 are included in Supplementary Dataset 2 in the column named "edgetic_disruptions". Each row in this column contains a list of 0's and 1's which correspond to predictions of disruption or non-disruption of interaction between the protein carrying the mutation and each interaction partner listed in the adjacent column named "partners". A value 0 under column "edgetic_disruptions" indicates that no disruption of interaction was predicted with the corresponding partner in the "partners" column, and a value 1 indicates that a disruption of interaction was predicted with the corresponding partner in the "partners" column.

In addition, we have added a new table in the manuscript (Table 1) that summarizes all the mutation edgotype data that was used to calculate the dispensable content among both transient PPIs and permanent PPIs in our two structural interactomes Y2H-SI and Lit-SI, for all different PPI properties (Page 26). We have also provided a second table (Table 2) that summarizes all the probabilities for effectively neutral mutations and mildly deleterious mutations to disrupt transient and permanent PPIs, for each type of PPI classification (Page 27). These probabilities in Table 2 were calculated directly from Table 1 by dividing the number of common mutations or disease mutations that disrupt a certain type of PPI by the total number of mutations as listed in Table 1, assuming that common mutations are effectively neutral and that disease mutations are mildly deleterious on average. These probabilities are also presented as pie charts in Figures 2-5, which were then used to directly calculate dispensable content among transient PPIs and permanent PPIs, as described in our example calculation in the Results section (Pages 11-14 Lines 201-254). Table 2 also includes the dispensable content calculated for transient PPIs and permanent PPIs based on each type of PPI classification described in the text. Hence, these two tables (Tables 1 and 2) in the revised version of the manuscript provide the reader with all the data needed to re-calculate all the dispensable content results presented in our Results section for transient and permanent PPIs.

Reviewer's Comments: (17) The authors talk about edgotype 'predictions' (lines 146 to 150) but isn't it more like a definition? First, there are no experimental evidences that the mutations impact the PPI (or that is discussed in ref 4 and therefore the authors should mention it). In ref 33, edgetic is

defined as loss of interactions, but in the table the mutation has modified the type of residue at an interfacial position and affected the ΔΔG by 0.5 kcal.mol^{-1} (data not found): is the FoldX 0.5 kcal.mol^{-1} enough to claim a loss of binding/interactions?

Author's Response: We thank the reviewer for the very important and constructive comment which helped us revise and improve our manuscript. We agree with the reviewer that not every mutation at the binding interface of a PPI will result in the complete disruption (loss) of the PPI. First, we would like to clarify that our definition of dispensable content among transient PPIs and permanent PPIs in human refers to those PPIs that are effectively neutral upon complete disruption by mutations, i.e., those PPIs whose complete elimination (loss) from the interactome has no deleterious impact on human fitness. Therefore, our goal in this study is to predict whether each PPI that carries a mutation at the binding interface is preserved in the presence of that mutation or completely eliminated from the interactome in the presence of that mutation. To do that, our structure-based approach predicts that a mutation at the PPI binding interface edgetically disrupts the PPI, i.e., eliminates it from the interactome, if and only if it causes a change in binding free energy ΔΔG > 0.5 kcal/mol. This ΔΔG cut-off has been used and proven to provide high-quality predictions of PPI disruption by mutations in previous structural biology studies (Zhao *et al.* 2014 *PLOS Comput. Biol.* 10:e1003592; Cui *et al.* 2019 *Genes* 10:933; Ghadie and Xia 2019 *Nature Commun.* 10:3205; Ghadie and Xia 2021 *Front. Bioinf.* 1:690769).

We also agree with the reviewer that it is important to support our structure-based predictions of PPI disruptions by mutations with experimental evidence. In our recent study (Ghadie and Xia 2021 *Front. Bioinf.* 1:690769), we have validated our FoldX-based edgotype prediction method on the experimental data of Sahni *et al.* 2015 (*Cell* 161:647-60) using the same ΔΔG cut-off that was used in our current study for predicting edgetic PPI disruption by mutations at the binding interface, 0.5 kcal/mol. We obtained a true positive rate (TPR) of 0.3 and a false positive rate (FPR) of 0.04 ($p = 0.002$, two-sided Fisher's exact test). A TPR that is equal to FPR indicates that predictions are no better than random expectations. Our TPR is 7.5 times larger than our FPR, further confirming that our choice of ΔΔG cut-off for predicting edgetic PPI disruptions is highly reliable. In addition, our Bayesian framework for estimating dispensable content has also been shown to be robust to different choices of ΔΔG cut-off close to 0.5 kcal/mol, particularly 0.3 and 0.7 kcal/mol (Ghadie and Xia 2021 *Front. Bioinf.* 1:690769), further proving that a ΔΔG cut-off in the neighborhood of 0.5 kcal/mol is highly reliable.

Nonetheless, we agree with the reviewer that some strong interactions may require a change in binding energy significantly larger than our cut-off of 0.5 kcal/mol to be disrupted by a mutation. In such cases, a mutation that causes a change in binding energy that is only slightly larger than 0.5 kcal/mol may be falsely predicted by our computational approach to disrupt the PPI. It is worth noting here that common mutations tend to have smaller ΔΔG values compared to disease mutations, with an average of 0.33 and 0.28 kcal/mol for common mutations in Y2H-SI and Lit-SI, respectively, compared to 1.1 and 0.9 kcal/mol for disease mutations, respectively (Figure S2 in Supplementary Information). Therefore, using a higher ΔΔG cut-off for predicting edgetic mutations is more likely to reduce the proportion of neutral mutations that are edgetic by a larger fraction compared to deleterious mutations, resulting in estimates of dispensable PPI content that are even lower than our current estimates. Indeed, we repeated our edgotype predictions and re-calculated dispensable content among both weak transient PPIs and strong permanent PPIs using significantly higher ΔΔG cut-offs for predicting edgetic PPI disruption by mutations: 1, 2 and 3 kcal/mol, and our estimates of dispensable content among both transient PPIs and permanent PPIs remain below ~20% (Table S2 in Supplementary Information), proving that

our estimates of dispensable content are robust to different choices of ΔΔG cut-off for predicting edgetic PPI disruptions.

We have revised our manuscript accordingly to include these important reference citations and experimental validations for our choice of ΔΔG cut-off in the Discussion section (Pages 34-35 Lines 636-655; Pages 35-36 Lines 672-681).

<u>Reviewer's Comments:</u> (18) Widely used method appears ten times in the manuscript sometimes more than once in the same sentence. A method widely used does not guarantee its accuracy and does not change its limits. FoldX is used but has limitations compared to real experiments so only some impact can be 'assumed' using FoldX. The authors may want to backup the reliability of the free energy calculation using FoldX with references comparing experimental and FoldX results. The authors can also explain that given the data, FoldX is a good approximation, but acknowledge that when experiments are performed things might be different.

In discussion: The authors discuss the high quality of their approach by referring to their own work and at the same time mentioning widely used methods with no references, so maybe it will re-enforce their results if they refer to colleagues using for example FoldX and validating it the free energy calculation compared to experiments.

<u>Author's Response:</u> We thank the reviewer for the very important and constructive comment which helped us significantly improve our manuscript. We do agree with the reviewer that a method that is widely used does not guarantee its accuracy and does not change its limits. Therefore, in the revised version of the manuscript we have removed the term "widely used method" from both statements in which the method FoldX was mentioned (Page 10 Line 179; Page 35 Line 656). We also removed the term "widely" from other parts of the manuscript to minimize its occurrence.

We also agree with the reviewer that FoldX has limitations, and that it is important to back up our FoldX calculations of PPI binding free energy with experimental validations. Since we could not find any references that validate FoldX calculations of binding energy against experiments, we decided to perform the validations ourselves. Thus, we benchmarked our FoldX-based predictions of PPI binding free energy (ΔG) against experimental ΔG values obtained from the Integrated Protein-Protein Interaction Benchmarks Database (Vreven *et al.* 2015 *J. Mol. Biol.* 427:3031-41). We selected PPIs with experimental $\Delta G < -12$ kcal/mol ($K_d < 10^{-9}$ M) to represent strong permanent interactions and those with experimental $\Delta G > -8$ kcal/mol ($K_d > 10^{-6}$ M) to represent weak transient interactions (La *et al.* 2013 *Proteins: Struct. Func. Bioinf.* 81:805-18). We then predicted ΔG values for these two groups of PPIs using FoldX. As a result, we obtained a Pearson correlation coefficient of 0.33 between predicted and experimental ΔG ($p = 3.4 \times 10^{-4}$), indicating that our FoldX predictions of PPI binding free energy are high quality. Next, we predicted PPIs to be transient or permanent using ΔG values calculated by FoldX, and compared our predictions to PPI classifications based on experimental ΔG values. For predicting transient PPIs, we obtained a balanced accuracy of 68%, a precision of 90%, a true positive rate (TPR) of 0.4, and a false positive rate (FPR) of 0.03. A TPR that is equal to FPR indicates that predictions are no better than random expectations. Our TPR is 13.3 times larger than our FPR ($p = 7.2 \times 10^{-7}$, two-sided Fisher's exact test), further proving that our computational approach for predicting weak transient PPIs and strong permanent PPIs using FoldX-based ΔG calculations is very high-quality.

We have revised our manuscript accordingly to include this important experimental validation in the Discussion section (Pages 30-31 Lines 547-565).

<u>Reviewer's Comments:</u> (19) Lines: Finally, our study reveals the power of structural and systems biology computations in elucidating the phenotypic effects of interactome network perturbations by genetic mutations. => This link between genotype and phenotype relies on FoldX in silico mutations and free energy calculations, PDB structural modeling, change in interfaces with no experimental validations, and many assumptions (interface mutation and free energy changes for edgetic assessment, neutral are common, mildly deleterious are disease-related). The authors could discuss the limit of FoldX and discuss ref 4 results to show to what extent a 'computational approach' is performing well compared to experiment. Are there experimental mutations in PPI of the two interactomes that do not show edgetic output but were predicted to do so by FoldX (or just by assuming a mutation at interface modify the binding)? Are there experimental mutations in PPI of the two interactomes that are edgetic as predicted by FoldX? As FoldX does not reproduce an experimental mutation, it is expected to find discrepancy between experiments and computational assessment of the dispensable PPI.

<u>Author's Response:</u> We thank the reviewer for the insightful and constructive comments which helped us revise and significantly improve our manuscript. The only large-scale, comprehensive mutation edgotyping experiments in the literature are performed by Sahni *et al.* (*Cell* 161:647-60). Unfortunately, a direct, head-to-head comparison between experiment and computation in this case is not possible, due to lack of high-quality structural models for the vast majority of PPIs that are disrupted by mutations in these experiments. That being said, all of the steps in our computational framework have been proven to be high quality and very reliable when benchmarked against multiple experimental datasets as described above in our previous responses.

To summarize, we have benchmarked our FoldX-based predictions of PPI binding free energy (ΔG) against experimental ΔG values. We obtained a Pearson correlation coefficient of 0.33 between predicted and experimental ΔG, indicating that our FoldX predictions of PPI binding free energy are high quality. Next, we predicted PPIs to be transient or permanent using ΔG values calculated by FoldX, and compared our predictions to PPI classifications based on experimental ΔG values. For predicting transient PPIs, we obtained a true positive rate (TPR) of 0.4 and a false positive rate (FPR) of 0.03. Our TPR is 13.3 times larger than our FPR ($p = 7.2 \times 10^{-7}$, two-sided Fisher's exact test), further proving that our computational approach for predicting weak transient PPIs and strong permanent PPIs using FoldX-based ΔG calculations is very high-quality.

Second, our FoldX-based method for predicting edgetic PPI disruption by mutations has been validated in our previous study on the experimental data of Sahni *et al.* 2015 (*Cell* 161:647-60), using the same $\Delta\Delta G$ cut-off we used in our current study for predicting edgetic PPI disruptions (Ghadie and Xia 2021 *Front. Bioinf.* 1:690769). Out of 23 mutations that were found to be edgetic in experiments, 7 were correctly predicted by our method to be edgetic, giving a true positive rate (TPR) of 0.3. On the other hand, out of 57 mutations that were found to be non-edgetic by experiments, only 2 were incorrectly predicted by our method to be edgetic, giving a false positive rate (FPR) of 0.04. Our TPR is 7.5 times larger than our FPR, indicating that our FoldX-based edgotype prediction method is very high quality as well.

At the same time, FoldX has been shown to provide high-quality predictions of binding $\Delta\Delta G$ in previous studies as well, with Pearson correlation coefficients ranging from 0.4 to 0.5 when benchmarked against comprehensive datasets of experimentally-determined $\Delta\Delta G$ values (Geng *et al.* 2019 *WIREs Comput. Mol. Science* 9:e1410). FoldX particularly outperforms other methods in identifying disease mutations (Gerasimavicius *et al.* 2020 *Scientific Reports* 10:15387). An independent experiment performed by our recent study (Ghadie and Xia 2019 *Nature Commun.* 10:3205) reported Pearson correlation coefficients of 0.5 and 0.42 for $\Delta\Delta G$ values predicted by

FoldX on co-crystal structures and homology models, respectively, when benchmarked against experimental data in the SKEMPI database (Jankauskaitė *et al.* 2019 *Bioinformatics* 35:462-9), further proving the high quality of FoldX-based calculations on PPI homology models.

Third, we demonstrated that our estimates of dispensable content among transient PPIs and among permanent PPIs are both robust to different choices of ΔΔG cut-off for predicting edgetic PPI disruptions, remaining below ~20% for all cut-offs. Fourth, we validated the high quality of our estimates of dispensable content derived from predicted mutation edgotypes in Y2H-SI and Lit-SI against experimental data, by re-calculating dispensable content among both transient PPIs and permanent PPIs using mutation edgotypes obtained from experiments of Sahni *et al.* (*Cell* 161:647-60). Overall, our estimates derived from experimental mutation edgotypes are consistent with our estimates derived from predictions. Taking all these experimental validations together, our study proves to be very high-quality and therefore greatly complements experimental efforts.

We have revised our manuscript accordingly to include all these reference citations and experimental validations demonstrating the high quality of our FoldX-based computational approach in the Discussion section (Pages 30-31 Lines 547-565; Pages 34-38 Lines 636-726).

RESPONSE TO COMMENTS FROM REVIEWER #2

<u>Reviewer's Comments:</u> (1) The authors present an analysis of the human interactome according to their dispensability and whether they're transient or permanent. The dispensability is estimated using a Bayesian framework the authors introduced in previous work and based on mapping different types of SNPs onto the transcriptome. Transient vs. permanent is investigated in a number of different ways, including interaction strength, transience in time and space, etc.. Their main finding is that dispensability is at <20% and broadly similar for transient/permanent interactions. It is an interesting and counterintuitive finding (see below). Overall, I think this is an interesting study, making use of fairly recently published large-scale data. A number of points to be addressed:

<u>Author's Response:</u> We thank the reviewer for the positive evaluation of our work and for the insightful and constructive comments which helped us significantly improve our manuscript.

<u>Reviewer's Comments:</u> (2) The result that neither interaction strength nor permanence in time/space (or mutual exclusivity) is a factor for dispensability is quite counterintuitive and perhaps should be discussed more, in particular from an evolutionary standpoint. One would think that selection pressure would be lower on several kinds of transient interactions, e.g., those between protein domains from recently much expanded families and its partner, than on very old and tight protein complexes.

<u>Author's Response:</u> We thank the reviewer for the very important comment which helped us revise and improve our manuscript. While our estimate of dispensable content among transient PPIs suggests that most transient PPIs in the human structural interactome carry out important cellular functions and are at least mildly deleterious upon disruption, this estimate represents an average over all transient PPIs in the structural interactome and is likely to vary significantly across the entire human interactome. For example, dispensable content may be higher among transient PPIs mediated specifically by motif-domain interactions in intrinsically disordered regions (Landry *et al.* 2009. *Trends Genet.* 25:193-7; Levy *et al.* 2012 *Phil. Trans. Roy. Soc. B Biol. Sci.* 367:2594-606). Selective pressures may also be lower among transient PPIs mediated by protein domains from recently much expanded families compared to PPIs mediated by more conserved domains

(Wan *et al.* 2015 *Nature* 525:339-44). While our study is only concerned about estimating the average fraction of dispensable PPIs among all transient PPIs in the human structural interactome, it remains to be seen whether the dispensable content varies significantly between these different groups of transient PPIs in the entire human interactome. We have revised our manuscript accordingly to include this important point in the Discussion section (Page 41 Lines 789-800).

Reviewer's Comments: (3) Also, if they were fully dispensable, there should also be fixed mutations (in chimp/mouse/etc.) that would interfere with these interactions?

Author's Response: We thank the reviewer for this very important comment which also helped us revise and improve our manuscript. The unique defining feature of completely dispensable PPIs is that their disruption has no measurable impact on organismal fitness. The reviewer correctly points out that PPIs that are completely dispensable across closely related species are expected to be less likely to be conserved across these species. That being said, PPIs that are completely dispensable in human may not be dispensable in other closely related species such as chimp and mouse, and as such they may or may not be conserved among closely related species. With currently incomplete PPI experimental datasets (with high false negative rates), it is hard to determine whether a specific PPI that is dispensable in human is conserved or not in another species. Nonetheless, recent genome-wide screens suggest that ~50% of PPIs in human are lost in budding yeast (Vo *et al.* 2016 *Cell* 164:310-23). Our current study suggests that at most ~20% of PPIs are completely dispensable in human (for both transient and permanent PPIs). Taken together, these results suggest that at least ~30% of PPIs are rewired between human and yeast not because they are unimportant in both species, but rather due to other factors such as lineage-specific adaption. While it has been shown that transient PPIs are more likely to rewire during evolution than permanent PPIs (Kim *et al.* 2006 *Science* 314:1938; Mintseris *et al.* 2005 *Proteins: Struc. Func. Bioinf.* 53:629-39; Beltrao *et al.* 2007 *PLOS Comput. Biol.* 3:e25; Sun *et al.* 2012 *PLOS Comput. Biol.* 8:e1002411; Studer *et al.* 2016 *Science* 354:229), these rewiring events may be driven by many complex evolutionary forces, including both non-adaptive (e.g., genetic drift on dispensable PPIs) and adaptive (e.g., lineage-specific adaptation) processes. Thus, the relationship between dispensability and evolutionary rate is a complex and significant research topic in and of itself, previously only investigated for proteins (Hirsh and Fraser 2001 *Nature* 411:1046-9; Pal *et al.* 2003 *Nature* 421:496-7; Hirsh and Fraser 2003 *Nature* 421:497-8), but never for PPIs. While our current study is focused on PPI dispensability in human, the relationship between dispensability and evolutionary rate for PPIs should be further investigated in future work. We have revised our manuscript accordingly to include this important discussion point in the Discussion section (Pages 41-42 Lines 800-818).

Reviewer's Comments: (4) I can see that there is relatively little data on Kds for PPIs, but I'm not sure how reliable FoldX is for absolute Kds. Perhaps at least a bit of benchmarking would be in order?

Author's Response: We thank the reviewer for this very important and constructive comment which helped us significantly improve our manuscript. We agree with the reviewer that FoldX has limitations, and that it is important to back up our FoldX calculations of PPI binding free energy with experimental validations. Thus, we benchmarked our FoldX-based predictions of PPI binding free energy (ΔG) against experimental ΔG values obtained from the Integrated Protein-Protein Interaction Benchmarks Database (Vreven *et al.* 2015 *J. Mol. Biol.* 427:3031-41). We selected

PPIs with experimental $\Delta G < -12$ kcal/mol ($K_d < 10^{-9}$ M) to represent strong permanent interactions and those with experimental $\Delta G > -8$ kcal/mol ($K_d > 10^{-6}$ M) to represent weak transient interactions (La *et al.* 2013 *Proteins: Struct. Func. Bioinf.* 81:805-18). We then predicted ΔG values for these two groups of PPIs using FoldX. As a result, we obtained a Pearson correlation coefficient of 0.33 between predicted and experimental ΔG ($p = 3.4 \times 10^{-4}$), indicating that our FoldX predictions of PPI binding free energy are high quality. Next, we predicted PPIs to be transient or permanent using ΔG values calculated by FoldX, and compared our predictions to PPI classifications based on experimental ΔG values. For predicting transient PPIs, we obtained a balanced accuracy of 68%, a precision of 90%, a true positive rate (TPR) of 0.4, and a false positive rate (FPR) of 0.03. A TPR that is equal to FPR indicates that predictions are no better than random expectations. Our TPR is 13.3 times larger than our FPR ($p = 7.2 \times 10^{-7}$, two-sided Fisher's exact test), further proving that our computational approach for predicting weak transient PPIs and strong permanent PPIs using FoldX-based ΔG calculations is very high-quality.

We have revised our manuscript accordingly to include this important experimental validation in the Discussion section (Pages 30-31 Lines 547-565).

<u>Reviewer's Comments:</u> (5) Along similar lines, a ddG of 0.5 seems to be a fairly low cut-off. In particular if a ΔG of -25 is used as the cut-off for "strong". Would it not mean that most "strong" interactions are hardly affected by some of the mutations? (going from, say, -30 kcal/mol to -29.5 kcal/mol).

<u>Author's Response:</u> We thank the reviewer for the very important and constructive comment which helped us significantly improve our manuscript. Our structure-based edgotype prediction approach predicts that a mutation at the binding interface edgetically disrupts the PPI if and only if it causes a change in binding free energy $\Delta\Delta G > 0.5$ kcal/mol. This ΔΔG cut-off has been used and proven to provide high-quality predictions of PPI disruption in previous structural biology studies (Zhao *et al.* 2014 *PLOS Comput. Biol.* 10:e1003592; Cui *et al.* 2019 *Genes* 10:933; Ghadie and Xia 2019 *Nature Commun.* 10:3205; Ghadie and Xia 2021 *Front. Bioinf.* 1:690769). In our recent study in particular (Ghadie and Xia 2021 *Front. Bioinf.* 1:690769), we have validated our FoldX-based edgotype prediction method on the experimental data of Sahni *et al.* 2015 (*Cell* 161:647-60) using a ΔΔG cut-off of 0.5 kcal/mol for predicting edgetic PPI disruption by mutation. We obtained a true positive rate (TPR) of 0.3 and a false positive rate (FPR) of 0.04. A TPR that is equal to FPR indicates that predictions are no better than random expectations. Our TPR is 7.5 times larger than our FPR ($p = 0.002$, two-sided Fisher's exact test), indicating that our FoldX-based edgotype prediction method and choice of ΔΔG cut-off for edgetic mutations is very high quality.

Nonetheless, we agree with the reviewer that some strong interactions may require a change in binding energy significantly larger than our cut-off of 0.5 kcal/mol to be disrupted by a mutation. In such cases, a mutation that causes a change in binding energy that is only slightly larger than 0.5 kcal/mol may be falsely predicted by our computational approach to disrupt the PPI. It is worth noting here that common mutations tend to have smaller ΔΔG values compared to disease mutations, with an average of 0.33 and 0.28 kcal/mol for common mutations in Y2H-SI and Lit-SI, respectively, compared to 1.1 and 0.9 kcal/mol for disease mutations, respectively (Figure S2 in Supplementary Information). Therefore, using a higher ΔΔG cut-off for predicting edgetic mutations is more likely to reduce the proportion of neutral mutations that are edgetic by a larger fraction compared to deleterious mutations, resulting in estimates of dispensable PPI content that are even lower than our current estimates. Indeed, we repeated our edgotype

predictions and re-calculated dispensable content among both weak transient PPIs and strong permanent PPIs using significantly higher ΔΔG cut-offs for predicting edgetic PPI disruption by mutations: 1, 2 and 3 kcal/mol, and our estimates of dispensable content among both transient PPIs and permanent PPIs remain below ~20% (Table S2 in Supplementary Information), proving that our estimates of dispensable content are robust to different choices of ΔΔG cut-off for predicting edgetic PPI disruptions by mutation.

We have revised our manuscript accordingly to include these important reference citations as well as experimental and computational validations that strongly justify our choice of ΔΔG cut-off in the Discussion section (Pages 34-35 Lines 636-655; Pages 35-36 Lines 672-681).

RESPONSE TO COMMENTS FROM REVIEWER #3

Reviewer's Comments: (1) Ghadie and Xia analyze the dispensable content among the transient protein-protein interactions (PPIs) by comparing them to permanent PPIs. They map PPIs to the structural protein interactome and consider them together with the neutral and disease-associated mutations. The overarching aim of the study is stated as assessment of the importance of transient PPIs quantitatively. For this aim, authors collect data from several resources, including high-throughput PPI data, tissue specific expression data, time series data and integrate them to label interactions as transient and permanent. Additionally, they compare these two interaction types based on several characteristics. Although the study has important analysis addition, I have major concerns on the novelty that should be addressed in the manuscript. I have divided my comments into the sections to easily follow.

Author's Response: We thank the reviewer for the positive evaluation of our work and for the insightful and constructive comments which helped us significantly improve our manuscript.

Reviewer's Comments: (2) Introduction: Introduction section has a very limited number of references despite there are many works studying transient interactions both computationally and experimentally. For example, between line numbers 53-65 authors have many strong statements, but none of them are supported with any citation. These statements need to be justified and properly supported with references.

Author's Response: We thank the reviewer for the very important comment which helped us revise and improve our manuscript. Indeed many studies have explored the structural, biophysical and spatiotemporal properties of transient and permanent PPIs (Nooren *et al.* 2003 *EMBO J.* 22:3486-92; Acuner Ozbabacan *et al.* 2011 *Protein Eng. Des. Sel.* 24:635-48; Aloy *et al.* 2004 *Nat. Biotechnol.* 22:1317-21). Studies have shown that the binding patterns of transient and permanent PPIs are largely controlled by the strength of interaction as well as co-expression of interaction partners (Jansen *et al.* 2002 *Genome Res.* 12:37-46; Han *et al.* 2004 *Nature* 430:88-93; Perkins *et al.* 2010 *Structure* 18:1233-43). A PPI is transient in time if the two interaction partners form a weak interaction only for a short period of time and then break apart (Nooren *et al.* 2003 *EMBO J.* 22:3486-92; Acuner Ozbabacan *et al.* 2011 *Protein Eng. Des. Sel.* 24:635-48; Perkins *et al.* 2010 *Structure* 18:1233-43). On the other hand, a PPI is permanent in time if the two interaction partners form a strong interaction that continues to exist without breaking apart thus forming a permanent protein complex (Nooren *et al.* 2003 *EMBO J.* 22:3486-92; Perkins *et al.* 2010 *Structure* 18:1233-43). Other studies have shown that PPIs can also be transient or permanent in space (Greene *et al.* 2015 *Nat. Genet.* 47:569-76). A PPI is permanent in space if the two interaction partners always

co-express to form stoichiometric complexes in the same tissues or cell types, and transient otherwise (Greene *et al.* 2015 *Nat. Genet.* 47:569-76; Marsh *et al.* 2015 *Nat. Commun.* 6:6394).

At the same time, transient PPIs differ from permanent PPIs in their structural composition and binding dynamics (Acuner Ozbabacan *et al.* 2011 *Protein Eng. Des. Sel.* 24:635-48; Perkins *et al.* 2010 *Structure* 18:1233-43; Vishwanath *et al.* 2017 *Curr. Opin. Struct. Biol.* 44:77-86). They tend to occur among certain protein hubs, known as "date hubs", which interact with multiple partners in a mutually exclusive manner using the same binding interface (Han *et al.* 2004 *Nature* 430:88-93; Kim *et al.* 2006 *Science* 314:1938). This behaviour is contrary to that of permanent PPIs which tend to occur among a different type of protein hubs, known as "party hubs", which interact with multiple partners simultaneously using multiple binding interfaces (Han *et al.* 2004 *Nature* 430:88-93; Kim *et al.* 2006 *Science* 314:1938). Mutually exclusive transient PPIs are often mediated through short linear motifs that typically occur in intrinsically disordered regions (Perkins *et al.* 2010 *Structure* 18:1233-43; Cumberworth *et al.* 2013 *Biochem. J.* 454:361-9; Tompa *et al.* 2014 *Mol. Cell* 55:161-9). These binding motifs tend to be smaller in surface area and contain less hydrophobic residues than interfaces of permanent PPIs, thus they bind with weaker affinities (Perkins *et al.* 2010 *Structure* 18:1233-43). Linear motifs also evolve very rapidly (Tompa *et al.* 2014 *Mol. Cell* 55:161-9; Perkins *et al.* 2010 *Structure* 18:1233-43), contributing in part to the higher rate of rewiring among transient PPIs compared to permanent PPIs (Mintseris *et al.* 2005 *Proteins: Struc. Func. Bioinf.* 53:629-39; Beltrao *et al.* 2007 *PLOS Comput. Biol.* 3:e25; Sun *et al.* 2012 *PLOS Comput. Biol.* 8:e1002411; Studer *et al.* 2016 *Science* 354:229). Indeed, empirical studies in phospho-proteomics and molecular evolution estimate that as much as ~65% of transient phosphorylation sites in yeast are unconstrained under evolution and may have no important function (Landry *et al.* 2009 *Trends Genet.* 25:193-7; Levy *et al.* 2012 *Phil. Trans. Roy. Soc. B Biol. Sci.* 367:2594-606).

We have revised our manuscript accordingly to include these important literature citations in the Introduction section (Page 4 Lines 48-58; Pages 5-6 Lines 81-94).

Reviewer's Comments: (3) "The question of how important transient PPIs......" sentence in line 62 sounds like still the importance of the transient PPIs is not discovered. However, many studies either experimentally or computationally demonstrated that transient PPIs are an important component of signaling pathways, membrane interactions. Additionally, many experimental works are present and going on to quantitively measure the transient PPIs. I would suggest authors to include a review of experimental efforts and why computational approaches are important in this sense.

Author's Response: We thank the reviewer for the very important and insightful comment which helped us significantly improve our manuscript. The question of how important transient PPIs are to cellular function is crucial to our understanding of cell systems biology and human disease (Vidal *et al.* 2011 *Cell* 144:986-98; Caldera *et al.* 2017 *Curr. Opin. Syst. Biol.* 3:88-94; Huttlin *et al.* 2017 *Nature* 545:505-9). Indeed, many transient PPIs have been found to play important roles in defining the structure of interactome networks, such as regulating interactome modularity (Han *et al.* 2004 *Nature* 430:88-93; Perkins *et al.* 2010 *Structure* 18:1233-43) and guiding the formation of obligate protein complexes (Blundell *et al.* 2006 *Nature* 444:279-80; Tang *et al.* 2006 *Nature* 444:383-6). In addition, as the reviewer mentioned, many transient PPIs are also known to participate in different cellular pathways and biochemical processes, including secretory pathways (Nyfeler *et al.* 2005 *PNAS* 102:6350), signal transduction (Sungkaworn *et al.* 2017 *Nature* 550:543-7; Thomas *et al.* 2004 *Nat. Rev. Neurosci.* 5:173-83; Kholodenko 2006 *Nat. Rev. Mol.*

Cell Biol. 7:165-76), immune response (Liu *et al.* 2007 *Nat. Rev. Immunol.* 7:202-12), chaperone-guided protein folding (Wu *et al.* 2019 *Nat. Commun.* 10:4833; Rosenzweig *et al.* 2019 *Nat. Rev. Mol. Cell Biol.* 20:665-80), apoptosis (Moll *et al.* 2005 *Curr. Opin. Cell Biol.* 17:631-6), and tumor suppression (Bieging *et al.* 2014 *Nat. Rev. Cancer* 14:359-70). While these studies show that many transient PPIs play important roles in cellular function and human disease, it remains unclear whether this observed functional significance generalizes to most transient PPIs.

Nevertheless, detecting transient PPIs in experiments and through computational predictions is very challenging (Acuner Ozbabacan *et al.* 2011 *Prot. Engin. Design Selec.* 24:635-48; Schilder *et al.* 2013 *Curr. Opin. Struct. Biol.* 23:911-8; Vishwanath *et al.* 2017 *Curr. Opin. Struct. Biol.* 44:77-86). With different limitations and biases associated with experimental techniques for detecting PPIs (Cafarelli *et al.* 2017 *Curr. Opin. Struct. Biol.* 44:201-10; Wodak *et al.* 2013 *Curr. Opin. Struct. Biol.* 23:941-53), large-scale datasets of transient PPIs are currently not available. However, databases that curate PPI sequence and structural information such as linear motifs (Kumar *et al.* 2020 *Nucleic Acids Res.* 48:D296-306; Grant *et al.* 2011 *Bioinf.* 27:1017-8), domain-motif interactions (Obenauer *et al.* 2003 *Nucleic Acids Res.* 31:3635-41), domain-domain interactions (Mosca *et al.* 2014 *Nucleic Acids Res.* 42:D374-9), three-dimensional protein structures (Berman *et al.* 2003 *Nature Struct. Mol. Biol.* 10:980), and binding affinities (Vreven *et al.* 2015 *J. Mol. Biol.* 427:3031-41) from both experimental and computational studies can be used to predict transient and permanent PPIs. Some studies have used PPI binding affinity measurements from experiments to identify transient and permanent interactions (Vreven *et al.* 2015 *J. Mol. Biol.* 427:3031-41; La *et al.* 2013 *Proteins: Struct. Func. Bioinf.* 81:805-18). The scope of these studies is limited by the small number of PPIs with affinity data available from experiments. Computational studies have used interface structural information (Mintseris *et al.* 2003 *Proteins: Struct. Func. Bioinf.* 53:629-39; Kim *et al.* 2006 *Science* 314:1938), protein sequence information (Ofran *et al.* 2003 *J. Mol. Biol.* 325:377-87) and machine learning models (Block *et al.* 2006 *Proteins: Struct. Func. Bioinf.* 65:607-22; Park *et al.* 2009 *BMC Bioinf.* 10:36) to predict transient and permanent interactions. Other studies made use of gene expression data which does not rely on experimentally-solved protein structures (Jansen *et al.* 2002 *Genome Res.* 12:37-46; Bossi *et al.* 2009 *Mol. Syst. Biol.* 5:260). Since the criteria and information that were used to detect transient interactions vary among these studies, each study has its own biases and sources of error. Moreover, with the large increase in PPI datasets recently mapped by experiments in human (Luck *et al.* 2017 *Trends Biochem. Sci.* 42:342-54; Luck *et al.* 2020 *Nature* 580:402-8), and given the difficulty in identifying transient PPIs in experiments, there is a great need for new computational efforts to accurately classify transient and permanent interactions in these new datasets, taking into account different structural and biophysical properties that distinguish transient PPIs from permanent PPIs.

We have also revised our manuscript accordingly to include this important discussion of previous experimental and computational work on transient PPIs and why there is a need for new computational efforts in the Introduction section (Page 5 Lines 70-81; Pages 6-7 Lines 95-113).

<u>Reviewer's Comments:</u> (4) In the last paragraph of the Introduction, authors summarize their study. However, it is very long, and many technical details are included which are already there in the Results. This makes the paragraph very diffuse. Additionally, authors describe how they constructed the structural interactome between lines 85-90 which is the same procedure of a work by the same authors (Ghadie and Xia 2019, Nat Comm.). Repeating this part reduces the novelty aspect of the work. Is there any significant difference, update or improvement in the methodology in the construction of the structural interactome in this manuscript compared to the previous one?

If yes, that aspect should be described. If no, I would suggest just citing their study and stating that the structural interactome in Ghadie and Xia 2019, Nat Comm. was used. I will have additional questions about the structural interactome in the other sections.

Author's Response: We thank the reviewer for this very important comment which helped us revise and improve our manuscript. We agree with the reviewer that some details mentioned in the last paragraph of the Introduction may not be necessary to include since they are also described in the Results section. Therefore, in the revised version of the manuscript, we have removed some of the details that we believe do not need to be explained in the Introduction. This includes the description of different properties that were used in our study to distinguish transient PPIs from permanent PPIs, as well as other technical details about constructing the human structural interactome. At the same time, while the procedure we used to construct the human structural interactome in this study is similar to that in Ghadie and Xia 2019, there are two major differences between this study and our previous study that cannot be highlighted in the Introduction without mentioning the main steps in our methodology. First, the human structural interactome constructed in this study is much larger than the structural interactome constructed in Ghadie and Xia 2019. Second, unlike in Ghadie and Xia 2019, in this study the edgotype of a mutation is defined separately for each group of PPIs, transient and permanent, which requires us to mention the main steps of predicting mutation edgotypes. Therefore, we have revised the last paragraph in the Introduction to better highlight what steps in our current methodology are similar to that in Ghadie and Xia 2019 and what steps are different. Our methodology described in the Introduction now reads as follows:

"Here, in an effort to answer the long-standing question of the overall importance of transient PPIs, we provide a quantitative measure of their importance and compare them with permanent PPIs by estimating dispensable content among both types of PPIs, using the same procedure we developed before to estimate dispensable content in the overall human interactome (Ghadie *et al.*, *Curr. Opin. Struc. Biol.* 50:42-48, 2018; Ghadie and Xia 2019 *Nature Commun.* 10:3205). Starting with a high-quality human reference interactome mapped by experiments, we apply homology modelling similar to Ghadie and Xia 2019 to construct a high-resolution three-dimensional (3D) human structural interactome with PPI binding interfaces annotated at the residue level (Figure 1a). This structural interactome is much larger than our previously constructed structural interactome in Ghadie and Xia 2019. Next, we label each PPI in the structural interactome as either transient or permanent based on different structural, biophysical and spatiotemporal properties (Figure 1a). We map common mutations from healthy individuals as well as Mendelian disease-causing mutations onto the human structural interactome and perform structure-based calculations similar to Ghadie and Xia 2019 to predict the edgotype for each mutation, i.e., the precise pattern of PPI perturbations as a result of each mutation. Unlike in Ghadie and Xia 2019, here the edgotype of a mutation is defined separately for each group of PPIs, transient and permanent. Finally, we integrate our edgotype predictions into the same Bayesian framework we used in Ghadie and Xia 2019 and calculate the fraction of PPIs that are completely dispensable, i.e., effectively neutral upon disruption by mutation, among each group of PPIs (Figure 1b)." (Page 7 Lines 114-131).

Reviewer's Comments: **(5) Results: The first two sections of the Results are a repetition of Ghadie and Xia 2019, Nat Comm. maybe with some dataset updates. My question is why the authors did not use the already available dataset that has been curated in their previous publication directly and label the transient and permanent PPIs but started the same pipeline from scratch and**

performed the same analysis to construct the structural interactome, interface extraction and mutation mapping again. The data is already there. I understand that there might be some updates in PPIs and datasets but still it will be an addition to the already available one.

Author's Response: We thank the reviewer for the very important question which helped us revise and improve our manuscript. As the reviewer mentioned, we had the option of using our older dataset for the human structural interactome and PPI perturbation predictions from our previous study in Ghadie and Xia 2019. The main reason we chose not to do so but instead constructed a new human structural interactome and predicted PPI perturbations again in this new interactome was to maximize the size of our dataset, which is essential for our current study. Unlike our previous study in Ghadie and Xia 2019 where we estimate dispensable content in the overall human interactome, in this study we estimate dispensable content among two subgroups of PPIs, transient and permanent, based on several structural and biophysical properties. Therefore, it is essential that we have a large coverage of PPIs and mutations in our structural interactome in order to obtain statistically meaningful estimates of dispensable content among both groups of PPIs.

The Y2H structural interactome (Y2H-SI) in our current study consists of 1,916 PPIs among 1,468 proteins (Supplementary Dataset 1A), and the literature-derived structural interactome (Lit-SI) consists of 4,676 PPIs among 3,445 proteins (Supplementary Dataset 1B). These two structural interactomes are much larger than the structural interactomes we had previously constructed in Ghadie and Xia 2019. Y2H-SI which was derived in our current study from the recent HuRI dataset (Luck *et al.* 2020 *Nature* 580:402-8) is 4 times larger in number of PPIs than the Y2H structural interactome in Ghadie and Xia 2019, which was derived from the much smaller HI-II-14 dataset (Rolland *et al.* 2014 *Cell* 159:1212-26). In addition, Lit-SI in our current study is also 1.4 times larger in number of PPIs than the literature-derived structural interactome in Ghadie and Xia 2019. As a result, Y2H-SI in our current study contains 1,428 mutations in total, which is 2.74 times the number of mutations (521) in Y2H-SI of Ghadie and Xia 2019. Similarly, Lit-SI in our current study contains 4,439 mutations in total, which is 1.34 times the number of mutations (3,302) in Lit-SI of Ghadie and Xia 2019. This significant increase in coverage in terms of PPI structural data and mutation data is essential for the statistical significance of our results. Notably, our estimates of dispensable content derived in this study from the HuRI dataset and the IntAct literature-curated dataset are consistent with our previous estimates in Ghadie and Xia 2019, which were derived from much smaller datasets.

We have revised our manuscript accordingly to highlight this difference in coverage between our two human structural interactomes (Page 9 Lines 158-162; Page 10 Lines 169-171; Page 29 Lines 515-519).

Reviewer's Comments: (6) The work classifies the permanent and transient interactions based on the calculated dG values of FoldX. How good is FoldX in predicting transient and permanent interactions? Finding a ground truth dataset having already known permanent and transient interactions and calculating the precision, recall and other performance measures would support that the labelled permanent and transient interactions are at some accuracy or precise.

Author's Response: We thank the reviewer for the very important and constructive comment which helped us revise and improve our manuscript. We agree with the reviewer that FoldX has limitations, and that it is important to back up our FoldX calculations of PPI binding free energy with experimental validations. Thus, we benchmarked our FoldX-based predictions of PPI binding free energy (ΔG) against experimental ΔG values obtained from the Integrated Protein-Protein Interaction Benchmarks Database (Vreven *et al.* 2015 *J. Mol. Biol.* 427:3031-41). We selected

PPIs with experimental ΔG < -12 kcal/mol ($K_d < 10^{-9}$ M) to represent strong permanent interactions and those with experimental ΔG > -8 kcal/mol ($K_d > 10^{-6}$ M) to represent weak transient interactions (La *et al.* 2013 *Proteins: Struct. Func. Bioinf.* 81:805-18). We then predicted ΔG values for these two groups of PPIs using FoldX. As a result, we obtained a Pearson correlation coefficient of 0.33 between predicted and experimental ΔG ($p = 3.4 \times 10^{-4}$), indicating that our FoldX predictions of PPI binding free energy are high quality. Next, we predicted PPIs to be transient or permanent using ΔG values calculated by FoldX, and compared our predictions to PPI classifications based on experimental ΔG values. For predicting transient PPIs, we obtained a balanced accuracy of 68%, a precision of 90%, a true positive rate (TPR) of 0.4, and a false positive rate (FPR) of 0.03. A TPR that is equal to FPR indicates that predictions are no better than random expectations. Our TPR is 13.3 times larger than our FPR ($p = 7.2 \times 10^{-7}$, two-sided Fisher's exact test), further proving that our computational approach for predicting weak transient PPIs and strong permanent PPIs using FoldX-based ΔG calculations is very high-quality.

We have revised our manuscript accordingly to include this important experimental validation in the Discussion section (Pages 30-31 Lines 547-565).

<u>Reviewer's Comments:</u> (7) My previous comment is also valid for the overall pipeline. The authors have some assumptions based on tissue- or temporal- expression to classify transient and permanent interactions. However, there is no validation on a set of already known transient and permanent interactions. Applying their classification approach to a benchmark dataset and evaluating their performance would make a solid basis for their classification. A relevant paper to find a benchmark dataset might be PMC4084939. There are more articles I presume cataloging a benchmark dataset.

<u>Author's Response:</u> We thank the reviewer for the very important and constructive comment which helped us significantly improve our manuscript. In addition to classifying PPIs based on strength of interaction, our study also classifies PPIs that are transient or permanent in time using three other properties: 1) low temporal co-expression for transient interaction partners versus high temporal co-expression for permanent interaction partners, 2) unbalanced stoichiometry for transient interactions versus balanced stoichiometry for permanent interactions, and 3) mutually exclusive binding for transient interactions versus simultaneously possible binding for permanent interactions. We agree with the reviewer that evaluating the performance of our approach for classifying transient and permanent interactions using a benchmark dataset will make a solid basis for our classifications. Thus, we validated our predictions of PPIs that are transient or permanent in time using the three properties mentioned above on a manually-curated dataset of transient and permanent complexes in human derived from two independent studies by La *et al.* 2013 (*Proteins: Struct. Funct. Bioinform.* 81:805-18) and Mintseris and Weng 2003 (*Proteins: Struct. Funct. Bioinform.* 53:629-39). For predicting transient PPIs based on temporal co-expression levels, we obtained a balanced accuracy of 70%, a precision of 99%, a TPR of 0.51, and a FPR of 0.1 (Table S1 in Supplementary Information). Our TPR in this case is 5.1 times larger than our FPR ($p = 0.019$, two-sided Fisher's exact test), indicating that our predictions of temporally transient PPIs are very high quality and much better than random expectations. For predicting transient PPIs based on stoichiometry, we obtained a balanced accuracy of 56%, a precision of 97% and a TPR to FPR ratio of 1.4, albeit with an insignificant p-value (Table S1 in Supplementary Information). Finally, by predicting mutually exclusive PPIs to be transient and simultaneously possible PPIs to be permanent, we obtained a balanced accuracy of 78%, a precision of 98%, a TPR of 0.95, and a FPR of 0.4 (Table S1 in Supplementary Information). A TPR to FPR ratio of 2.4 ($p = 1.1 \times 10^{-5}$,

two-sided Fisher's exact test) further proves that our computational approach for predicting temporally transient PPIs and temporally permanent PPIs is high-quality. Notably, a large part of PPI classifications in the benchmark dataset of La *et al.* were derived from experimental ΔG values using rigid cut-offs and may contain some errors. Therefore, the quality of our computational predictions may be even better than what is observed by validation on this dataset.

Unlike the case for PPIs that are transient or permanent in time, experimental benchmark datasets for PPIs that are transient or permanent in space are not available. This is because while transience in time can be accurately detected through protein 3D structure analysis and other single-cell data analysis, detecting transience in space across multiple body tissues and in different cell types is more challenging and time-consuming. Nonetheless, a previous computational study by Bossi and Lehner 2009 (*Mol. Syst. Biol.* 5:260) has also attempted to predict spatially transient and spatially permanent PPIs based on gene co-expression levels in 79 human body tissues. Unlike our study which classifies PPIs using gene expression data from the Illumina Body Map 2.0 project and the Fantom5 project, the study of Bossi and Lehner classified PPIs using gene expression data from the GNF Atlas project (Su *et al.* 2004 *PNAS* 101:6062). While such dataset may not be accurate enough to be considered a gold standard for benchmarking our predictions of transient and permanent PPIs, it provides an independent set of PPI classifications that can be used to validate our estimates of dispensable content among spatially transient and spatially permanent PPIs. Thus, we repeated our calculations of dispensable content, this time labelling spatially transient and spatially permanent PPIs using the dataset of Bossi and Lehner. We considered a PPI to be permanent in space if it was predicted by Bossi and Lehner to exist in at least 90% of the tissues, otherwise we considered the PPI to be transient in space. In Lit-SI, we estimated that ~6% of spatially transient PPIs are completely dispensable with a 95% confidence interval of ~3-11%, and that ~8% of spatially permanent PPIs are completely dispensable with a 95% confidence interval of ~3-19%. These estimates remain below 20% in agreement with our previous estimates. A similar calculation of dispensable content in the recently mapped interactome Y2H-SI was not possible due to lack of PPI classifications in the dataset of Bossi and Lehner, which only includes PPIs that were published in the literature at the time of their study.

We have revised our manuscript accordingly to include these important experimental and computational validations in the Discussion section (Pages 31-33 Lines 566-611).

<u>Reviewer's Comments:</u> (8) Authors collect the expression levels from GEO times series datasets and Illumina Body Map tissue expression dataset. More information needs to be added about these datasets. Are the samples from healthy people or from a patient? Are there the mutation profiles available for these samples in the same data sources? If yes, how much overlap is present between the sample mutation profiles and the mutations collected from dbSNP and ClinVar? The follow-up question would be how this information would affect their analysis, if available?

<u>Author's Response:</u> We thank the reviewer for the insightful comment which helped us revise and improve our manuscript. Our study uses gene tissue expression data from the Illumina Body Map and Fantom5 projects to classify PPIs as spatially transient or spatially permanent. This expression data was measured in normal human body tissues. Our study also uses gene time-course expression data from the GEO database to classify PPIs as temporally transient or temporally permanent. This expression data was measured in different cell types under varying conditions. While it is very difficult to obtain detailed information about the health condition of subjects participating in these projects, it is possible that some gene expression levels may vary for individuals with certain diseases and in certain clinical settings, which may possibly introduce some errors into our

classifications of transient and permanent PPIs. It is also possible that expression levels of some genes may be affected by mutations carried by the same gene.

Our study assumes that gene expression samples were obtained from healthy individuals, which is true for the Illumina Body Map dataset and the Fantom5 dataset, but may not always be true for GEO data. Our study also assumes that the expression level of a gene is not affected by mutations in the gene itself. We address these limitations by considering other biophysical and structural properties of PPIs that are independent from gene expression levels, such as the strength of interaction and the number of mutually exclusive PPIs sharing the same binding interface. The consistency of our estimates of dispensable content using different PPI properties proves that our computational framework is robust to such possible sources of error. Furthermore, the high quality of our PPI classifications and estimates of dispensable PPI content as demonstrated by validations against multiple independent experimental and computational datasets of transient and permanent PPIs further proves the robustness of our study to such possible errors in PPI classifications. Unfortunately, mutation profiles associated with the gene expression samples that were used in our study are not provided by the database. However, sample information provided in our supplementary files may be used in the future for further investigation of mutation impact on gene expression.

We have revised our manuscript accordingly to include this important discussion in the Discussion section (Pages 33-34 Lines 612-635).

Reviewer's Comments: (9) I think the novelty aspect of the study is hidden in the expression-based classification of permanent and transient interactions. However, interestingly, this aspect is not presented in the current manuscript as much as it deserves. Actually, Table 1 summarizes their overall results, but it is rather orphan in the text and not explained in detail in the Result section to give a detailed understanding about the results.

Author's Response: We thank the reviewer for the very important comment which helped us revise and improve our manuscript. Indeed, not many studies have used gene expression data to classify transient and permanent PPIs. Some studies have used PPI binding affinity measurements from experiments to identify transient and permanent interactions (Vreven *et al.* 2015 *J. Mol. Biol.* 427:3031-41; La *et al.* 2013 *Proteins: Struct. Func. Bioinf.* 81:805-18). The scope of these studies is limited by the small number of PPIs with affinity data available from experiments. Computational studies have used interface structural information (Mintseris *et al.* 2003 *Proteins: Struct. Func. Bioinf.* 53:629-39; Kim *et al.* 2006 *Science* 314:1938), protein sequence information (Ofran *et al.* 2003 *J. Mol. Biol.* 325:377-87) and machine learning models (Block *et al.* 2006 *Proteins: Struct. Func. Bioinf.* 65:607-22; Park *et al.* 2009 *BMC Bioinf.* 10:36) to predict transient and permanent interactions. Only a few studies made use of gene expression data which does not rely on experimentally-solved protein structures (Jansen *et al.* 2002 *Genome Res.* 12:37-46; Bossi *et al.* 2009 *Mol. Syst. Biol.* 5:260). Since the criteria and information that were used to detect transient interactions vary among these studies, each study has its own biases and sources of error. Moreover, with the large increase in PPI datasets recently mapped by experiments in human (Luck *et al.* 2017 *Trends Biochem. Sci.* 42:342-54; Luck *et al.* 2020 *Nature* 580:402-8), and given the difficulty in identifying transient PPIs in experiments, there is a great need for new computational efforts to accurately classify transient and permanent interactions in these new datasets, taking into account different structural and biophysical properties that distinguish transient PPIs from permanent PPIs.

Our study provides a comprehensive list of transient PPIs and permanent PPIs in human, characterized using diverse structural and biophysical properties. These properties include, among others, the temporal and spatial co-expression of interaction partners, a property that has not been explored enough in recent literature especially on large-scale PPI datasets such as the HuRI dataset. While discrete properties such as the number of mutually exclusive PPIs sharing the same binding interface can be strong indicators of the transient or permanent nature of interaction, other properties such as binding affinity and the spatial and temporal co-expression of interaction partners may vary for some PPIs over a continuum of values overlapping between transient PPIs and permanent PPIs. For example, while transient PPIs tend to have weak binding affinities, it may be that some of them have stronger binding affinities closer to those of permanent PPIs. At the same time, while transient PPIs tend to have low co-expression levels and unbalanced stoichiometries between interaction partners, some of them may have higher co-expression levels and more balanced stoichiometries closer to those of permanent PPIs. Hence, there is no perfect borderline that separates transient PPIs from permanent PPIs based on such properties. Our study addresses this limitation by considering different structural, biophysical and spatiotemporal properties for classifying transient and permanent interactions. These properties produce different sets of PPI classifications that are diverse enough to capture the aforementioned variabilities in PPI properties, but also with significant overlap (Supplementary Dataset 5). On average, a PPI is classified as transient by ~4 out of 8 properties in both Y2H-SI and Lit-SI, with 36% of PPIs in Y2H-SI and 40% of PPIs in Lit-SI classified as transient by 5 or more properties (Figure S1 in Supplementary Information). This diversity in PPI classifications results in a noticeable change across different PPI properties in the proportions P(T|N), P(T|M), P(P|N) and P(P|M) of neutral (N) and mildly deleterious (M) mutations that edgetically disrupt transient (T) and permanent (P) PPIs (Tables 1 and 2, Pages 26-27). Nonetheless, the two ratios of proportions P(T|N)/P(T|M) and P(P|N)/P(P|M) are less affected by such changes (Table 2, Page 27), resulting in estimates of dispensable PPI content that are broadly consistent across all PPI properties (<~20%) (Table 2, Page 27). These results highlight the importance of considering different biophysical measurements in our study for classifying transient PPIs and permanent PPIs.

We have revised our manuscript accordingly to better highlight this novelty aspect in using different measurements to classify transient and permanent PPIs in the Introduction (Pages 6-7 Lines 101-113) and Discussion (Pages 29-30 Lines 520-546).

We agree with the reviewer that it is important to provide the detailed calculation for our results shown in Table 1, which is now Table 2 in the revised manuscript. As suggested by the reviewer, in the revised manuscript, we have included the detailed calculation of dispensable content among weak transient PPIs and among strong permanent PPIs in the Y2H structural interactome in the Results section as an example calculation (Pages 11-14 Lines 201-254). All other calculations of dispensable content throughout the manuscript follow the exact same calculation provided in the Results section for weak and strong PPIs. In addition, we have also included a detailed description of our Bayesian framework for calculating dispensable content in the Methods section (Pages 46-47 Lines 902-923).

In addition, we have provided another table in the revised manuscript (Table 1) that summarizes all the mutation edgotype data that was used to calculate the dispensable content among each type of PPIs in the two structural interactomes Y2H-SI and Lit-SI (Page 26). We have also provided in Table 2 (Page 27) all the probabilities for effectively neutral mutations and mildly deleterious mutations to disrupt transient PPIs and permanent PPIs, for each type of PPI classification in Y2H-SI and Lit-SI. These probabilities in Table 2 were calculated directly from

Table 1 by dividing the number of mutations that disrupt a certain type of PPI by the total number of mutations as listed in Table 1. This calculation is also shown in our example calculation in the Results section (Pages 11-14 Lines 201-254). Table 2 also includes the dispensable content calculated for transient PPIs and permanent PPIs based on each type of PPI classification described in the text.

<u>Reviewer's Comments:</u> (10) Other Comments: Discussion section reads like an extended abstract of the manuscript. It needs a detailed rewriting and discussing the results based on available literature.

<u>Author's Response:</u> We thank the reviewer for all the insightful comments and suggestions which helped us significantly improve our manuscript. As described above in our previous responses to reviewer comments, the discussion section has been substantially expanded and improved in the revised version of the manuscript. In particular, we discussed in detail the strengths and limitations of our computational methods for classifying transient and permanent PPIs using different structural and biophysical properties. We validated these methods against multiple independent experimental and computational datasets, showing that our methods are very high quality. We also validated our FoldX-based method for predicting PPI affinities as well as PPI perturbations by mutations against experimental datasets, also showing that our FoldX-based prediction methods are very high quality as well. In addition, we provided solid justifications for our choice of prediction methods using available literature and computational simulations when needed. Furthermore, we discussed the strength and robustness of our estimates of dispensable PPI content to different choices of parameters and to possible computational errors by comparing them to experimental data available from the literature. Finally, we discussed the importance of our estimate of dispensable content among transient PPIs from an evolutionary perspective based on available literature. These discussion points have all been included in the Discussion section (Pages 28-38 Lines 506-726; Pages 40-42 Lines 774-818).

<u>Reviewer's Comments:</u> (11) I strongly encourage the authors to deposit all relevant codes to a repository and make it available for the reproducibility of the study.

<u>Author's Response:</u> We thank the reviewer for the important suggestion. We have made all relevant code available online. The code that was used to construct the human structural interactome is available at https://github.com/MohamedGhadie/build_structural_interactome. The code that was used to map mutations onto the human structural interactome and calculate dispensable content among transient PPIs and permanent PPIs is available at https://github.com/MohamedGhadie/dispensable_transient_ppis. We have included these links to our code in the Code Availability statement in the revised manuscript (Page 48).

<u>Reviewer's Comments:</u> (12) Author's summary section repeats the Abstract. It is expected to provide a non-technical summary of the work. I suggest reconsidering this aspect and revising the summary.

<u>Author's Response:</u> We thank the reviewer for the important suggestion. As requested, we have revised the author summary to minimize the use of technical terms and to make it accessible to all non-expert readers. The summary now reads as follows: "All cellular functions are driven by interactions between different biomolecules in the cell. Among these interactions are protein-

protein interactions, abbreviated as PPIs, in which two proteins physically bind to each other to perform a specific molecular function. PPIs are often divided into two categories: transient PPIs in which two proteins bind to each other only for a short time and then break apart, and permanent PPIs in which two proteins bind to each other permanently thus forming a permanent protein complex. Considering their permanent nature, it is generally assumed that permanent PPIs are important for cellular function, whereas it remains unclear whether transient PPIs are equally important. Here, we estimate the fractions of transient PPIs and permanent PPIs in human that can be removed from the cell without harming human fitness. We do this by constructing three-dimensional structural models for PPIs which allow us to predict the probabilities for disease-causing mutations and non-disease mutations from healthy individuals to disrupt transient PPIs and permanent PPIs. Using these probabilities, we estimate that similar to permanent PPIs only a small fraction of transient PPIs (<~20%) can be disrupted without harming fitness, indicating that most transient and permanent PPIs are important for cellular function in human." (Page 3).
